# Synthetic β-d-Glucuronides: Substrates for Exploring Glucuronide Degradation by Human Gut Bacteria

**DOI:** 10.1021/acsomega.4c09036

**Published:** 2024-12-20

**Authors:** Aleksandra Gorecka, Heidi Schacht, Megan K. Fraser, Aleksandra Teriosina, James A. London, Igor L. Barsukov, Andrew K. Powell, Alan Cartmell, Andrew V. Stachulski, Edwin A. Yates

**Affiliations:** † Department of Chemistry, University of Liverpool, Liverpool L69 7ZD, U.K.; ‡ Department of Biochemistry, Cell and Systems Biology, ISMIB, University of Liverpool, Crown Street, Liverpool L69 7ZB, U.K.; § School of Biological Sciences, University of Liverpool, Crown Street, Liverpool L69 7ZB, U.K.; ∥ School of Pharmacy and Biomolecular Sciences, Liverpool John Moores University, Byrom Street, Liverpool L3 3AF, U.K.; ⊥ Department of Biology, University of York, Heslington, York YO10 5DD, U.K.

## Abstract

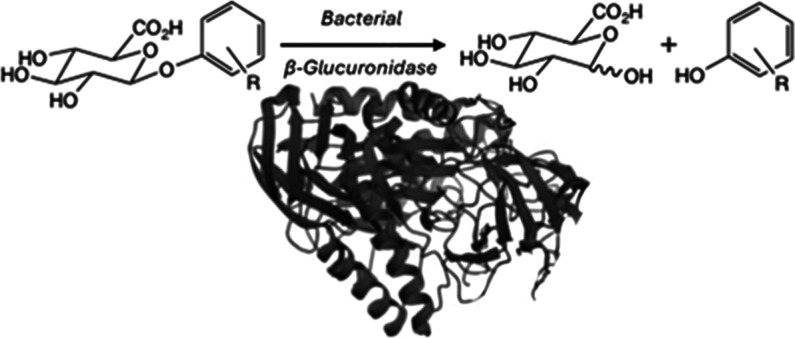

The human gut microbiota (HGM) is a complex ecosystem subtly dependent on the interplay between hundreds of bacterial species and numerous metabolites. Dietary phenols, whether ingested (e.g., plant-derived guaiacol, mequinol, or resveratrol) or products of bacterial fermentation (e.g., *p*-cresol), have been attributed with influencing bacterial growth and host health. They are cleared by phase II metabolism, one form utilizing β-d-glucuronidation, but encounter bacterially derived glucuronidases capable of hydrolyzing them to release their phenolic and glucuronic acid moieties with potential effects on host cells or the surrounding bacterial population. Tools to enable the detailed study of their activity are currently lacking. Syntheses of β-d-glucuronides from methyl 1,2,3,4 tetra-acetyl β-d-glucopyranosyluronate by direct glycosylation with 2-, 3-, or 4-methoxy- and 4-fluorophenol acceptors employing trimethylsilyl triflate catalysis are reported. Yields (methoxy series) were modest. An improved route from methyl 1,2,3,4-tetra-acetyl β-d-glucopyranosyluronate via selective anomeric deprotection (*N*-methyl piperazine) and conversion to an α-trichloroacetimidate glycosyl donor was employed. Coupling with 2- and 3-methoxyphenol acceptors and deprotection provided 2- and 3-methoxyphenyl β-d-glucuronides in 2-fold improved overall yield. These naturally occurring methoxyphenyl glucuronides augment available model substrates of dietary glucuronides, which include 3- and 4′-linked resveratrol. The use of model glucuronides as substrates was illustrated in studies of β-d-glucuronidase activity employing cell lysates of 9 species of HGM (*Bacteroidetes*), revealing distinct outcomes. Contrasting effects on bacterial growth were also observed between the free phenolic components, their respective glucuronides, and glucuronic acid. The glucuronide of 4-fluorophenol provided sensitive and background-free detection of β-glucuronidase activity using ^19^F NMR.

## Introduction

The human gut microbiota (HGM) is a complex ecosystem containing a large number of bacterial species engaged in complex association with each other and with the host.^1^ The microbiota influences, and potentially helps to regulate, numerous functions that include host immunity and the nervous system,^2^ while its microbial composition is linked to diet.^3^ Changes in microbiome composition occur over the lifetime of the host,^4^ reduced diversity being associated with aging, while more rapid changes occur through significant dietary changes,^5^ and are also influenced by social and environmental factors.^6^

Under certain conditions, such variations as well as infection and antibiotic use can modify the HGM composition, leading to dysbiosis and disease.^7,8^

One class of compounds that can influence the composition of the gut are dietary phenols, a major dietary source of which are the aromatic amino acids tyrosine and phenylalanine that undergo bacterial fermentation in the gut to produce the toxin, *para*-cresol (*p*-cresol; 4-methylphenol),^9−13^ but many other potentially toxic phenols can be released from food directly. Examples are the simple phenols 2-methoxyphenol (guaiacol) from fruit^14^ and 3- and 4-methoxyphenol (mequinol), both released from lignin and when food is wood-smoked. The latter pair of compounds are also used as food improvement agents. Examples of more complex phenols include the stilbene, resveratrol (3,5,4′-trihydroxy *cis*-/*trans*-stilbene), an antifungal and antibacterial phytoalexin found, for example, in nuts and grape skins, for which conflicting benefits to human health have been claimed.^15^ Nevertheless, whatever the health benefits of individual compounds may be, phenols can be toxic. One facet of the toxicity of *p*-cresol, for example, resides in its ability to cause DNA damage resulting in cell-cycle arrest,^16^ and disruption of the cell cycle in colonic epithelial cells has also been reported.^17^

The clearance of *p*-cresol, other dietary phenols, as well as numerous xenobiotics from the body is achieved primarily through phase-II metabolism via sulfation and glucuronidation,^18^ both of which increase solubility and accelerate excretion.^19^ The former involves the enzymatic addition of a sulfate group, and the resulting products are the subject of increasing scrutiny owing to their effects on the host and on the microbiome.^20,21^ The latter, which is the focus of the present paper, is achieved through the enzymatic addition of a β-linked d-glucuronic acid (GlcA) moiety by one of a family of UDP glucuronosyltransferases (UGTs)^22^ that, while occurring predominantly in the liver,^19^ can also be carried out by other cells including intestinal enterocytes.^23^ Furthermore, a number of UGT enzymes that are absent from the liver have been identified in the intestine.^24^ There is, therefore, the possibility that phenyl β-d-glucuronides, biosynthesized by the host in the intestine, could come into contact with bacterially derived β-d-glucuronidases (GUS) which, in addition to hydrolyzing xenobiotics,^18^ could also rerelease their glucuronic acid and phenolic moieties.^1,25^ What the subsequent fates of the products of the hydrolysis of these dietary glucuronides are remains uncertain, but possibilities include their direct metabolism, absorption, or binding (leading, for example, to effects on host cell inflammation) serving either as a resource for, or inhibitors of, competing bacterial growth or as disruptors of bacterial quorum sensing.^26,27^

The major polysaccharide degrading bacterial genus of the HGM, *Bacteroides* (*Bacteroidota*) are considered good indicators of gut health.^28^ Among the many potential interactions between bacterial species in the human gut microbiome, the ability of *Bacteroides fragilis* to correct infections caused by *Clostridioides difficile* in a mouse model has been reported,^29^ while *Bacteroides thetaiotaomicron* has been shown to attenuate *C. difficile* colonization, as well as having several other beneficial effects on the host.^30^ The mechanisms underlying these mutual effects are currently not well-understood. One potential point of contact between *Bacteroides* and *Clostridioides* are dietary glucuronides and their phenolic constituents, although the relationship and its consequences are likely to be complex. For example, numerous bacterial strains across diverse families produce *p*-cresol,^31^ and this may bestow an advantage in the case of *C. difficile*,^32^ while treatment of mice with *p*-cresol was found to increase the levels of *p*-cresol-producing commensal bacteria as well as to alter the behavior of the mice.^33^ As examples of bacteria possessing glucuronidase activity, *Bacteroides uniformis* expresses three GUS enzymes and can hydrolyze both polysaccharides and small molecule glucuronides,^34^ while *C. difficile* is able to metabolize phenylalanine via *para*-hydroxyphenylacetic acid to *para*-cresol.^35^ Analysis of the carbohydrate active enzyme database reveals further examples of bacteria from the HGM that possess glucuronidase capabilities including *Clostridium perfringens*, *Escherichia coli* K12, *Eubacterium eligens*, *Faecalibacterium prausnitzii,* and many *Bacteroides* species.^36^ The ability of these bacteria to degrade different glucuronides and the relative susceptibility of bacterial species to the various phenolic compounds released are also not fully known, but it will be important to understand these nuanced interactions in detail if we are to begin to unravel the complex processes mediated by these compounds within the HGM. Currently, authentic glucuronide substrates with which to assess their hydrolysis are lacking, and we rely on model substrates such as *p*-nitrophenyl β-d-glucuronide. It is also important to develop this understanding because it has been proposed that the levels of phenols such as *p*-cresol in feces may provide a biomarker of *C. difficile* infection.^37^ If other interactions and processes are involved, these could impact the level of such metabolites, compromising their effectiveness as biomarkers. Although the levels of other phenolic dietary compounds such as methoxyphenols including guaiacol (2-methoxyphenol) and mequinol (4-methoxyphenol) or polyphenols such as resveratrol (*cis*/*trans*-3,5,4′-trihydroxystilbene) are unlikely, normally, to rival those of *p*-cresol derived from the ingestion of protein-rich foods, they may still exercise significant effects on both host cells and bacteria of the HGM.

Following our investigation into the synthesis and activities of methylphenyl β-d-glucuronides (cresyl glucuronides), their effects on mammalian cells,^38^ and the synthesis of resveratrol β-d-glucuronides,^39^ we report the preparation of the *o*-, *m*-, and *p*-methoxyphenyl β-d-glucuronide series and a 4-fluorophenyl β-d-glucuronide, which provides a useful ^19^F NMR signal for screening glucuronidase activity essentially background free in biological samples.

The methoxyphenyl glucuronide series and the resveratrol 3- and 4′-β-d-glucuronides recently described^39^ provide a useful panel with which to investigate glucuronide hydrolysis by the cell lysates of nine *Bacteroides* species from the HGM, as well as comparisons of the effects of the glucuronide and the potential products of hydrolysis—the respective phenic moieties and glucuronic acid—on bacterial growth to be made. 4-Methoxyphenol is licensed as a component in topical dermatological treatments,^40,41^ a food flavoring agent, and is also a component of insect repellent, solvents, and plastics. Human exposure to resveratrol, on the other hand, is mainly from the ingestion of nuts and fruit, but daily intake may exceed 100s of mgs, if taken as a dietary supplement.

The synthetic compound 4-fluorophenol is a component of several pharmaceuticals and agrochemicals including cisapride, which increases gastrointestinal contractions, and the neuroprotective agent sabeluzole, whose degradation involves the formation of glucuronides,^42^ and also occurs in agrochemicals including herbicides, used to induce desiccation in broad-leaved weeds. Incorporation of fluorine into agrochemicals has increased by 40% since 2016,^43^ and the exposure of the HGM to such synthetic phenols also seems destined to increase. The synthetic approach described here was also applied to 4-fluorophenyl β-d-glucuronide, allowing its potential degradation by cell lysates of the panel of bacterial species from the HGM to be monitored using ^19^F NMR, providing a further illustration of the versatility of model glucuronides.

## Results and Discussion

### Synthesis of *o*-, *m*-, and *p*-Methoxyphenyl β-d-Glucuronides

The synthesis of the full series comprising *o*-, *m*-, and *p*-methoxyphenyl β-d-glucuronides has not, to the best of our knowledge, been reported, although some of these glucuronides and their protected precursors are known. Thus, the *o*-methoxy ester intermediate and final glucuronide have been documented,^41^ and the ester intermediate of the 4-methoxy derivative, prepared via a forerunner of the direct method reported here from the β-anomeric tetra-ester, has also been made.^44^ The syntheses of the full series and, where absent, full characterization are reported herein. The NMR data for all compounds are included (see Supporting Information) to provide a convenient set of data for reference.

### Preparation of the *o*-, *m*-, and *p*-Methoxyphenyl β-d-Glucuronides via a Direct Glycosylation Strategy

**Scheme 1:**
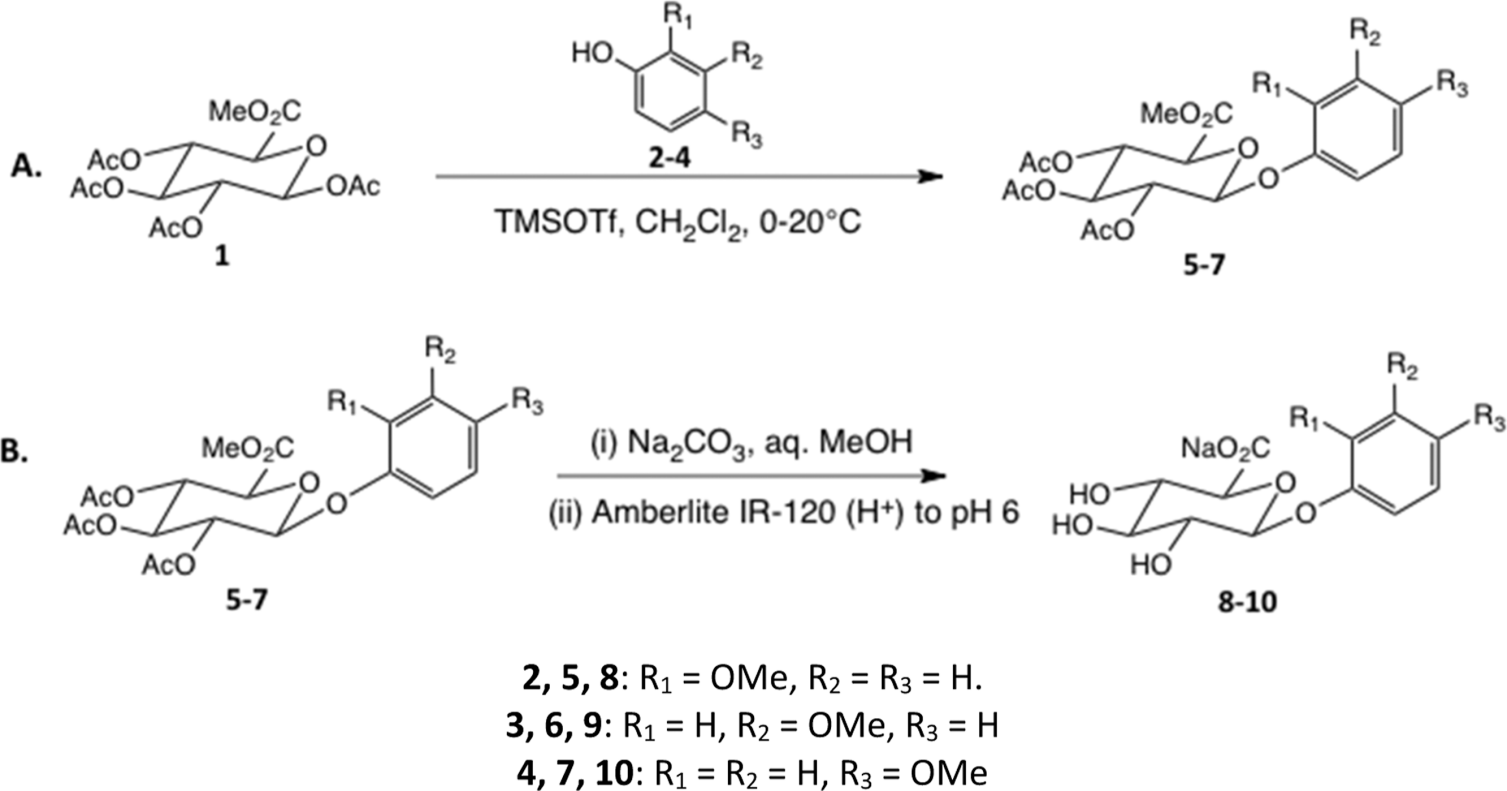
Direct Syntheses of β-d-Glucuronides of the *o*-, *m*-, and *p*-Methoxyphenyl Series by Analogy with the Cresyl Series:^38,39^ (A) Glycosylation of the Methoxyphenols to Afford Protected Glucuronides, 24–38%; (B) Hydrolysis Affording Final Products as Na Salts, 53–71% See Supporting Information for additional details.

**Scheme 2:**
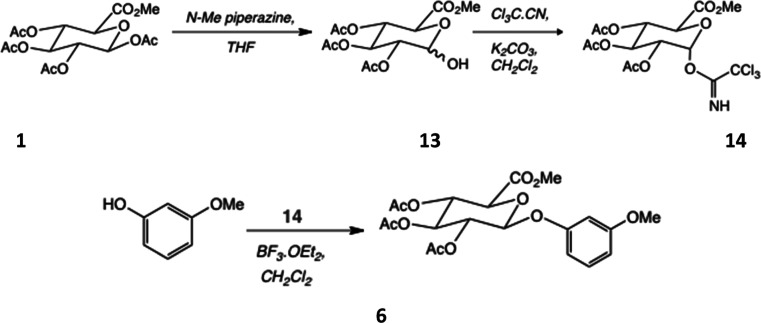
Synthesis of a Glucuronide Intermediate Using the Imidate Method

**Scheme 3:**
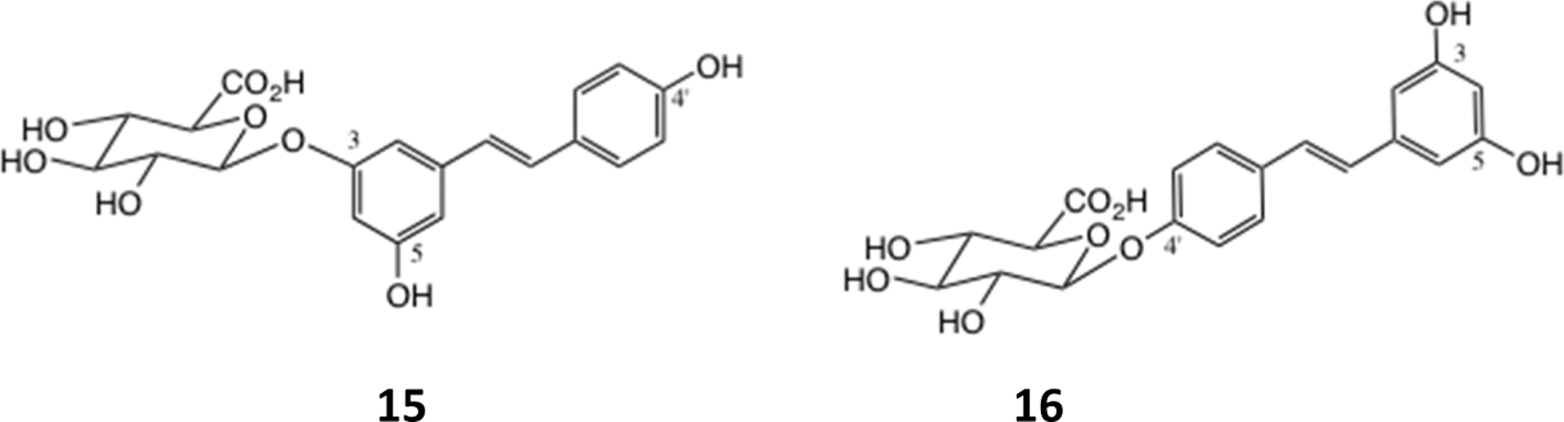
Resveratrol 3-*O* (**15**) and 4′-*O* (**16**) β-d-Glucuronides^39^

Initially, the synthetic route analogous to that used for the β-d-cresyl glucuronides via the fully protected methyl-1,2,3,4-tetra-*O*-acetyl-β-d-glucopyranuronate (**1**) and the appropriate methoxyphenol acceptor employing TMS triflate catalyst (Scheme 1) under nitrogen established by London et al.^38^ was attempted. Following a mild deprotection step involving simultaneous hydrolysis of acetyl and methyl esters, this provided the target compounds in modest yields (24–38%) [Scheme 1; compounds (**8**), (**9**), and (**10**)]. Following purification, as an example, the *p*-methoxyphenyl form (**10**) was employed, together with other available model glucuronides of resveratrol (**15**) and (**16**) (Schemes 2 and 3), in further investigations of the ability of bacteria of the human gut to hydrolyze them.

### Preparation of the Imidate Glycosyl Donor Was via Selective Deprotection of the 1-*O*-Acetyl Group

Owing to the modest yields obtained with the direct glycosylation approach outlined above, we also explored an alternative pathway (Scheme 2) that involved the formation of the imidate glycosyl donor intermediate (**14**) as a potentially efficient route to a β-d-glucuronide from the commercially available methyl-1,2,3,4-tri-*O*-acetyl-β-d-glucopyranuronate (**1**) as the starting material.^6^ For this route to prove feasible, a suitable base capable of selectively deprotecting the anomeric acetate without disturbing any other acetate group or the methyl ester functionality to generate (**13**) had to be used. A number of reagents have been used (summarized in ref (39)) and were explored. Briefly, with ammonium acetate (p*K*_a_ 9.9), the reaction did not occur, even when left overnight; NMR of the crude products showed no change compared to the starting material. An overnight reaction with morpholine (p*K*_a_ 8.36) was then tried, which cleaved the targeted acetyl group while also producing several byproducts, not all of which were separable by column chromatography; the final yield was a disappointing 29%. During the course of these investigations, however, we did discover that *N*-methylpiperazine (NMP) (p*K*_a_ 9.4) gave excellent results.^39^ Thus, treatment of (**1**) with NMP buffered by AcOH, in THF or acetonitrile, gave a 90% yield of (**13**) (Scheme 2) by simple solvent extraction at pH 3.

The ^1^H NMR spectrum of the hemiacetal (**13**)^45,46^ showed a mixture of α and β anomers, ca. 5:1, with loss of the anomeric proton of the starting tetraester at 5.79 ppm. This intermediate was then converted into imidate (**14**)^47^ by reaction with trichloroacetonitrile and potassium carbonate in DCM under an inert atmosphere; NaH from the original method was replaced as the weaker base K_2_CO_3_ was known to be sufficient.^48^ Overnight reaction allowed complete conversion to the α-anomer (**14**) in 89% yield (thermodynamic product), confirmed by analysis of ^1^H–^1^H coupling constants by NMR: ^3^J_12_ = 3.6 Hz.

### Glucuronidation and Deprotection

Glucuronidation of the *o*-methoxyphenol acceptor (**2**) with the activated intermediate (**14**) was carried out in anhydrous DCM with BF_3_OEt_2_ catalysis.^49^ Such reactions exhibit complete selectivity for the β anomer, evinced by ^1^H–^1^H coupling constant analysis: e.g., ^3^J_12_ = 7.28 Hz for (**5**). The yield was more than twice as high as that obtained by tetraester coupling [58% compared to 24% for (**5**)]. Furthermore, the products were easily purified by column chromatography to afford the desired glucuronides, e.g., (**5**) in protected form. The protecting groups were cleaved using sodium carbonate in aqueous MeOH^38^ to generate the final products (**8**), isolated as the Na salts shown, and no free glucuronic acid was found in any of the samples by ^1^H NMR (the H5 resonance of the glucuronic acid moiety is shifted downfield ∼0.1 pm with respect to that of the sodium salt^39^). The final Na salt of the 4-methoxyphenyl glucuronide (**10**) was purified by recrystallization. Although this route was more time-consuming, it provided a purer final product with higher yield. The sodium salt has been reported as more stable over prolonged times, avoiding slow acid-catalyzed hydrolysis of the glycosidic bond.^39^

### Purification of Methoxyphenyl β-d-Glucuronides and Subsequent Characterization

Chromatography on silica, investigated by TLC, was unsatisfactory because of the high polarity of the final products. As an alternative, gel permeation chromatography (Biogel P2) was employed for the final purification step. Elution of the products from the GPC column in water was retarded unexpectedly, most likely due to hydrophobic interaction between the phenyl moiety of the glucuronides and the matrix material (polyacrylamide) but, nevertheless, GPC provided purified (**8**) which, following drying, yielded product suitable for subsequent investigations. Thin layer chromatography of the products (EtOAc/MeOH 1:1, v/v) provided single spots, and chromatography showed one major product (Figure S4).

**Figure 1:**
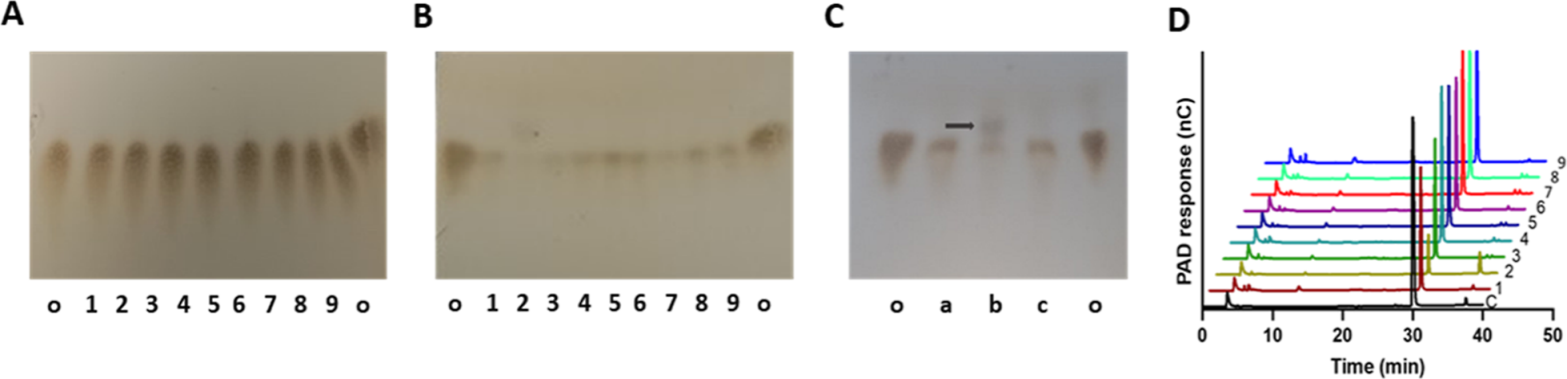
Bacteroides species of the HGM show differential hydrolysis of 4-methoxyphenyl β-d-glucuronide. (A–C) Thin-layer chromatograms (ethyl acetate/methanol, 1:1: v/v, 1 ascent, charring in concentrated sulfuric acid/ethanol, 1:9: v/v) showing 4-methoxyphenyl β-d-glucuronide incubated with cell lysates of 9 bacterial species from the HGM. Lanes marked o contain the glucuronide alone. (A) At the outset of the digestion, no hydrolysis of the glucuronide is evident in any of the lanes. (B) After 5 days incubation at 37 °C, all lanes show some evidence of digestion compared to control [lanes o, equal loading of material in (A,B)], but the extent of hydrolysis varies. In panels (A,B), species are as follows: lane 1, *Bacteroides caccae* (ATCC 43185); lane 2, *Bacteroides cellulosilyticus* (DSM 14838); lane 3, *Bacteroides clarus* (DSM22519/A 20/YIT 12056); lane 4, *Phocaeicola dorei*, formerly *Bacteroides* (DSM 17855); lane 5, *Bacteroides ovatus* (ATCC 8438); lane 6, *Bacteroidesintestinalis* (DSM 17393); lane 7, *Bacteroides salyersae* (DSM 18765); lane 8, *Bacteroies nordii* (CL02T12C05); and lane 9, *B. thetaiotaomicron* (VPI-5482)*.* (C) Thin-layer chromatogram with increased loading (x3) showing differential hydrolysis of 4-methoxyphenyl β-d-glucuronide following incubation with selected cell lysates of: a, *Bacteroides* (*Phocaeicola*) dorei (DSM 17855); b, *Bacteroides cellulosiyticus* (DSM 14838); and c, *Bacteroides intestinalis* (DSM 17393). Lanes marked o contain the untreated glucuronide at the initial concentration. (D) HPLC (electrochemical detection) of the same lysates of the 9 Bacteroides species incubated with 4-methoxyphenyl β-d-glucuronide (control, C, eluting at 30 min; reduced loading compared to lanes 1–9) reveals variable extent of degradation. The phenol moiety 4-methoxyphenol elutes in this system at 24.5 min and is not evident (by electrochemical or UV detection in the products; equal loading lanes 1–9), suggesting that it is largely metabolized. The bacteria corresponding to lanes a to c used in panel (C) are in lanes 4, 2, and 6, respectively, in panel (D).

**Figure 2:**
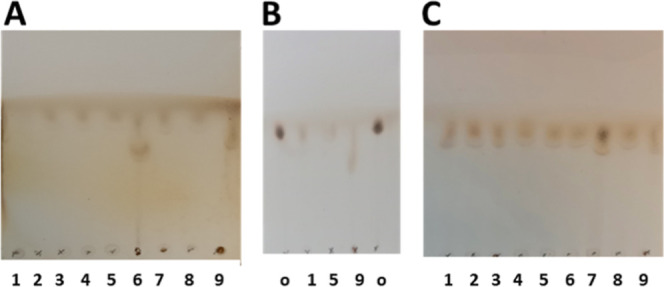
Thin-layer chromatograms (methanol, 1 ascent) showing resveratrol β-d-glucuronides treated with 9 bacterial lysates. (A) Resveratrol 3-*O*-β-d-glucuronide. Lanes 1–9 correspond to the 9 bacterial lysates. (B) A repeat of treatments using lysates 1, 5, and 9; lane marked o containing untreated glucuronide control. (C) Resveratrol 4′-*O*-β-d-glucuronide. Lanes 1–9 correspond to the 9 bacterial lysates.

**Figure 3:**
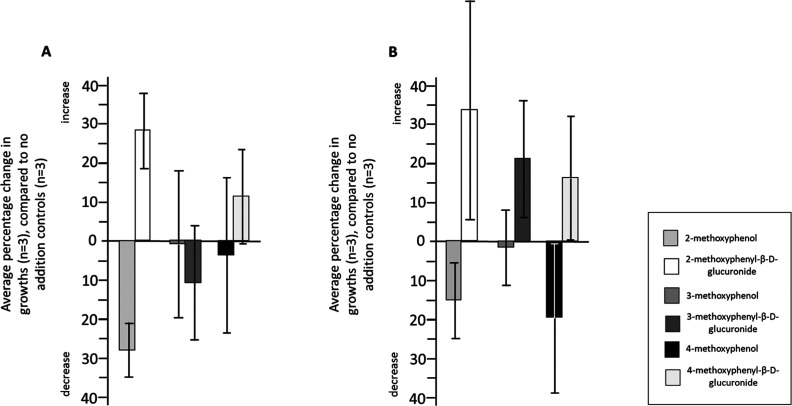
Examples of the varied effects of the 2-, 3-, 4-methoxyphenols **2**, **3**, and **4**, and their 2-, 3-, 4-methoxyphenyl glucuronide derivatives, **4**, **7**, and **10** on the growth of (A) *B. caccae* and (B) *B. thetaiotaomicron* in BHI media. Percentage changes report the average overall growth relative to the appropriate positive controls (*n* = 3). The bars represent ± error in percentage ratios (*A* ± d*A*/*B* ± d*B*), where *A* and *B* are average values and d*A* and d*B* are their respective standard deviations, combined according to error = [(d*A*/*A*)^2^ + (d*B*/*B*)^2^]^1/2^. Growth curves of triplicates, including negative and positive controls, are shown in Figures S31–S34.

Selected methoxyphenyl- and resveratrol glucuronides were then subjected to degradation by lysates of a panel of nine anaerobic bacteria (Figures 1 and 2), and the effects on bacterial growth of the free phenol forms and the corresponding glucuronide were also explored (Figure 3).

### Degradation of 4-Methoxyphenyl Glucuronide by Bacteroides

The ability of cell lysates from a panel of 9 representative *Bacteroidetes* species from the HGM to degrade the model glucuronide, 4-methoxyphenyl β d-glucuronide, was demonstrated using TLC (Figure 1A,B) and is shown for clarity (Figure 1C) with increased loading in 3 contrasting cases (lanes 4, 2, and 6 in Figure 1A and B, corresponding with lanes a, b, and c in Figure 1C). All 9 bacterial hydrolysates were also analyzed by HPAEC (Figure 1D). The extent of glucuronide breakdown and, in the case of (bacteria 2) *Bacteroides cellulosilyticus* (DSM 14838), also some of the products of digestion (Figure 1C, lane b) were distinct, although, despite a similar *R*_f_ to the phenolic moiety, 4-methoxyphenol (Figure S4), HPLC revealed that the products were not simply the released phenol, whose elution time (24.5 min; detected by UV) did not correspond to any of the peaks present in the products (Figure 1D). The charred products visible by TLC were not consistent with liberated glucuronic acid [whose *R*_f_ in ethyl acetate/methanol (1:1, v/v) is distinct; 0.12–0.49]. These results, supported by subsequent NMR analyses (see Figures S11 and S12), suggest that both the extent of hydrolysis and the degradative fate of glucuronides vary between lysates from distinct bacterial species.

### Comparison of the Effect of the Free Methoxy Phenols **2**, **3**, and **4** on Bacterial Cell Growth Compared to Their Glucuronides **4**, **7**, and **10**

The glucuronide series of the methoxyphenols, comprising **4**, **7** and **10**, enables activity comparisons to be made not only with each other, but also with the respective parent phenols, **2**, **3** and **4**. Growth curves were obtained in triplicate for two of the bacterial strains, *Bacteroides caccae* and *B. thetaiotaomicron* (bacteria 1 and 9 respectively), comparing overall growth with the respective positive controls in which no addition of phenol had been made. The relative increase or decrease in growth compared to the control, shown in Figure 3A,B reveals that the glucuronide form generally does not inhibit growth, while the phenol forms exhibit some inhibitory activity. Nevertheless, while there is seemingly a trend, with the majority of cases suggesting that the phenolic forms reduce growth and, that the glucuronide forms increase growth, only in the case of the 2-methoxy derivatives with *B. caccae* (Figure 3A) are those differences statistically significant (*p* < 0.01). It may be of potential interest that, as was the case for the activity of the cresol series and their glucuronides,^38^ the 2-methoxy-(*ortho*-) substituted form exhibited activity. The analysis of specific phenols and glucuronides on individual bacterial species has been little studied, still less their effects on an ensemble of bacteria or the human gut microbiome, but the compounds reported here will form the basis of future studies in this area.

### Degradation of Resveratrol 3-*O*- and 4′-*O*-β-d-Glucuronides by Bacteroides

The *Bacteroides* cell lysates were also tested with both the resveratrol 3-*O*- and 4′-*O*-glucuronides (Figure 2). Resveratrol 3-*O*-glucuronide was partially hydrolyzed by all species tested but, notably more by *Bacteroides intestinalis* (DSM 17393) and *B. thetaiotaomicron* (VPI-5482) (lanes 6 and 9 in Figure 1A) and a new, as yet, unidentified product with lower *R*_f_ was also evident in these lanes. The result for *B. thetaiotaomicron* (lane 9) is shown, comparing lanes 1, 5 and 9, together with untreated glucuronide (lane o) (Figure 1B). The effect of the lysates on resveratrol 4′-*O*-glucuronide (Figure 1C) was again, partial degradation, although less efficiently in the case of *Bacteroides salyersae* (DSM 17855) (Figure 2C, lane 7).

A control experiment, comprising a TLC run with lysates of bacteria 1–9 with no glucuronides added (Figure S2) confirmed that the migrating charred products on TLC in both Figures 1 and 2 derive ultimately from the glucuronide and not from the bacterial cell culture. The lower running products visible in Figure 2A lanes 6 and 9, and Figure 2B, lane 9, ran with a similar *R*_f_ to resveratrol (*R*_f_ in methanol, 0.74) but not with glucuronic acid (*R*_f_ in methanol, 0.32–0.58). Subsequent ^1^H NMR experiments (Figure S3) demonstrated that lower-running products isolated from TLC did not exhibit ^1^H NMR signals consistent with either E- or Z-forms of free resveratrol (or its cyclized form, phenanthrene)^50^ but did exhibit signals of additional products as well as some remaining resveratrol glucuronide (E-isomer). If resveratrol were released by hydrolysis, it will inhibit bacterial growth as observed for *B. thetaiotaomicron* (an average 86% reduction in overall growth, *p* < 0.01, *n* = 3), while the glucuronide form showed no significant inhibitory effect. Example growth curves under anaerobic conditions in the presence of resveratrol 3-*O*-glucuronide and resveratrol are shown in Figures S35 and S36.

### Effect on Bacterial Growth of GlcA in Minimal Media

**Figure 4:**
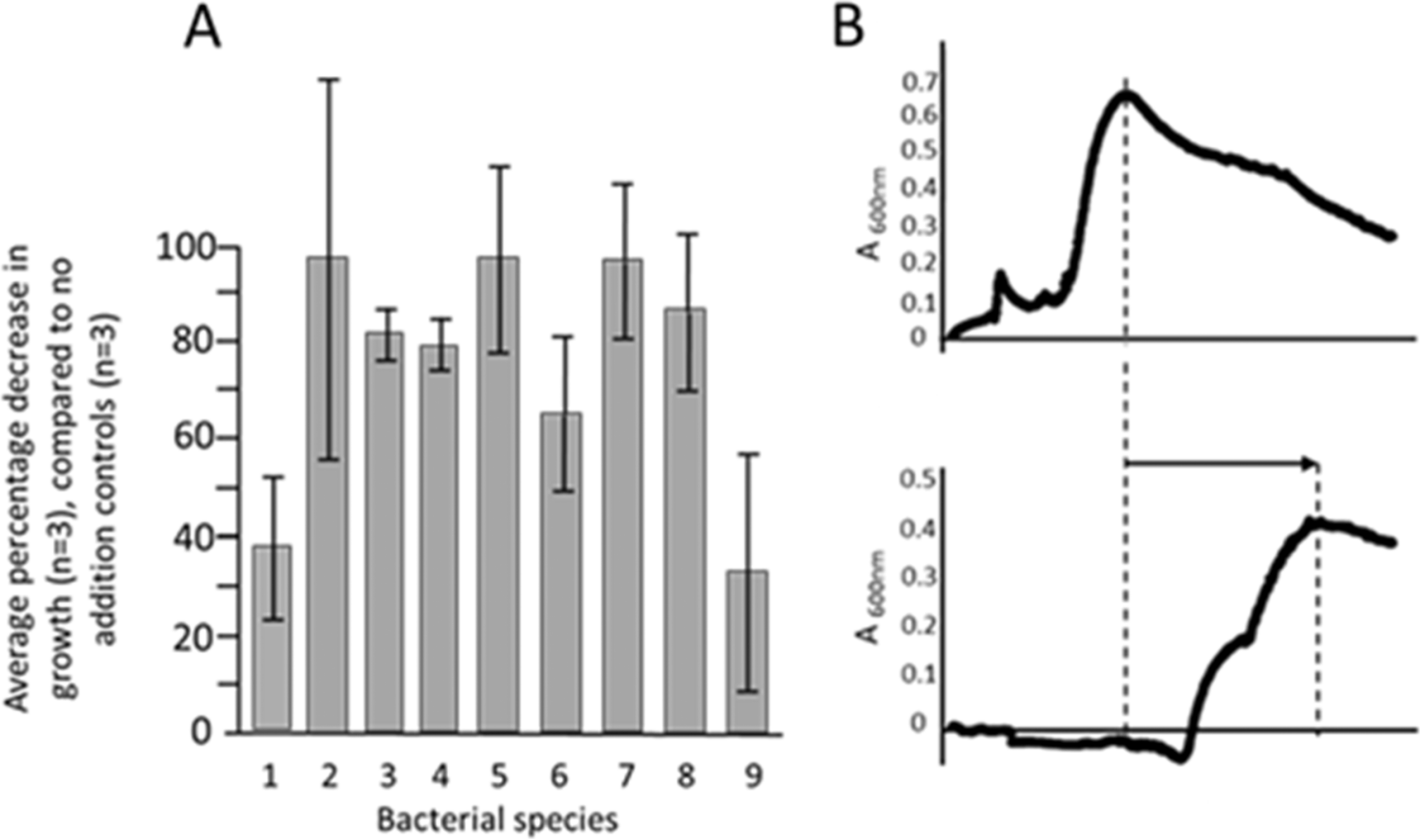
A) Effect of glucuronic acid (1 mM) on the growth of nine bacterial species under anaerobic conditions in minimal media. Percentage decrease in the average overall bacterial growth relative to the appropriate positive (no addition of GlcA) controls (*n* = 3). Bacterial species: 1, *B. caccae*; 2, *B. cellulosilyticus*; 3, *B. clarus*; 4, *P. dorei*; 5, *B. ovatus*; 6, *B. intestinalis*; 7, *B. salyerse*; 8, *B. nordii*; and 9, *B. thetaiotaomicron.* The bars represent ± error in percentage ratios (*A* ± d*A*/*B* ± d*B*), where *A* and *B* are average values and d*A* and d*B* are their respective standard deviations, combined according to error= [(d*A*/*A*)^2^ + (d*B*/*B*)^2^]^1/2^. (B) Representative growth curves over 48 h in minimal media for bacterium 3, *B. clarus*; (upper) control in the absence of glucuronic acid, (lower) in the presence of 1 mM glucuronic acid. The growth is delayed, and its extent is reduced in the presence of glucuronic acid. Growth curves are provided in Figure S37.

The hydrolysis of glucuronides also liberates GlcA in addition to the parent phenol, and its effect on bacterial growth (addition of 1 mmol to minimal media without glucose) was also ascertained for the nine bacterial species compared to positive controls (minimal media with glucose but no addition of glucuronic acid), employing growth curves obtained under anaerobic conditions. In all cases, the growth of the bacteria was attenuated compared to the respective positive control (Figure 4), although considerable variation was observed among the bacterial species; *B. caccae* and *B. thetaiotaomicron* being least affected. Addition of 1 mM glucuronic acid to minimal media (Figures 4A,B and S31) in the absence of glucose caused a delay in growth for all nine species and a decrease in overall growth for at least five species: 1, 3, 4, 8, and 9 (as an example, the effect on species 3, *Bacteroides clarus*, is shown in Figure 4B). The remainder, species 2, 5, 6, and 7, had not completed the exponential growth phase by the end of the recording period (48 h) (Figure S37).

**Scheme 4:**
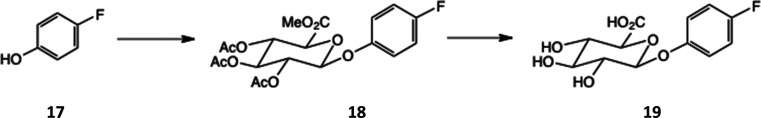
Synthesis of 4-Fluorophenyl β-d-Glucuronide (**19**) The sequence of chemical steps is as shown in Scheme 2.

### Monitoring the Degradation of 4-Fluorophenyl β-d-Glucuronide by Bacteroides Using ^19^F NMR

**Figure 5:**
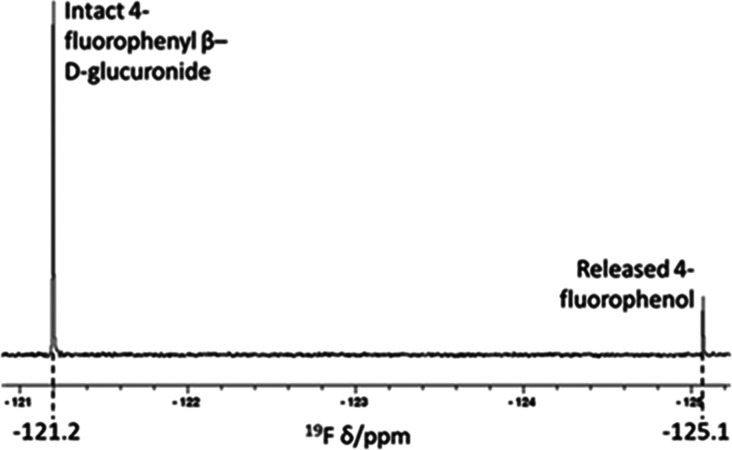
^19^F NMR monitoring of 4-fluorophenyl β-d-glucuronide hydrolysis to release 4-fluorophenol (−125.1 ppm) by the most active bacterial lysate (from *B. nordii*) with reference to the intact glucuronide (−121.2 ppm).

The synthetic approach was also used to prepare the unnatural glucuronide 4-fluorophenol (**17**) (Scheme 4). Either the trichloroacetimidate or the β-tetraester method (Schemes 1 and 2 above) was viable here; the former method afforded glucuronide ester **18** in 69% yield and hydrolysis gave the desired glucuronide **19** in excellent yield as its Na salt. The presence of the fluorine atom provided the opportunity of following hydrolysis using ^19^F NMR, which has high sensitivity, and is capable of either confirming that the free phenol is released from the glucuronide (an example is shown in Figure 5), or of detecting potential structural changes in the products by dint of the new chemical shift position of the ^19^F NMR signal. Bacterial lysates from the same panel of 9 *Bacteroides* species employed above were prepared and incubated with samples of (**19**) in PBS overnight.

Interestingly, all of the bacterial cell lysates exhibited some ability to degrade (**19**), summarized in Table 1, although none showed evidence of additional fluorine-containing products being produced, which would be evident from additional peaks. ^19^F NMR proved capable of detecting even low levels of degradation (<1%) with ease (Table 1) and essentially free of background signals (Figure 5). A fluorine-containing glucuronide such as (**19**) may provide a convenient tool for the detection of β-glucuronidase activity in samples whose complex background signals may obscure the detection of glucuronide and/or any potential products when using conventional detection systems.

**Table 1:** Percentage Degradation of 4-Fluorophenyl β-d-Glucuronide by Cell Lysates of Bacterial Species Detected by ^19^F NMR

bacterial species	percentage free phenol
B. caccae	12
B. cellulosilyticus	8
B. clarus	7.5
P. dorei (formerly Bacteroides)	9
B. ovatus	10
B. intestinalis	2.5
B. salyersae	<1
B. nordii	10
B. thetaiotaomicron	2

## Discussion

The synthesis of the (*o*-, *m*-, and *p*-) methoxyphenyl β-d-glucuronide series, based on direct glycosylation of a fully protected glucuronic acid derivative (**1**) using TMS triflate catalysis, has been reported. Improved yields for the 2- and 3-methoxyphenyl derivative were obtained by employing a longer synthetic route than that developed for the analogous cresyl glucuronide series, which had exploited direct glycosylation of the fully protected derivative, methyl 1, 2,3,4 tetra-*O*-acetyl d-glucuronate and TMS triflate catalysis.^38^ This alternative synthetic route (Scheme 2) employed partial hydrolysis of the same starting material (**1**) to yield the 2,3,4 tri-*O*-acetyl, methylester d-glucuronate (**13**) using *N*-methyl piperazine to achieve selective deacetylation of the anomeric acetate, followed by formation of the imidate glycosyl donor (**14**) with α-anomeric configuration (the thermodynamic product). This employed an adaptation of the original Schmidt imidation procedure^45,46^ in which NaH is replaced by the milder and safer K_2_CO_3_.^48^ Glycosyl coupling of 2-methoxyphenol (**2**) with (**14**) was achieved using BF_3_·OEt_2_ or TMSOTf catalysis, affording (**5**) in a 58% yield. The product (**5**) was then deprotected in mild basic conditions, viz., aq. Na_2_CO_3_/MeOH, to remove both the remaining acetyl groups and to cleave the methyl ester, affording the glucuronide (**8**). The selective deacetylation at the anomeric position and generation of α-configured imidate glycosyl donor intermediate allowed efficient coupling, affording the desired β-product in a higher yield [27% (4 steps) vs 13% (2 steps)] for this longer synthetic route.

Central questions regarding the HGM include the nature of relationships between diet and host nutrient availability and population balance, the influence that species have on each other, and the principles underlying the interactions between them. In beginning to address these questions, it is first useful to identify any potential molecular species that involve, or are produced by, different bacterial species and also the molecules through which such mutual effects could be exercised. One such class are the dietary phenols and their respective glucuronides, the synthesis of some which we have reported previously,^38,39^ and additional examples are provided here. Attempts to address questions around the interplay of phenolic compounds, their glucuronide derivatives, and bacteria of the HGM are in their infancy and will, no doubt, be assisted greatly by a variety of the so-called “omics” approaches. Nevertheless, the model substrates reported here can be employed to test the ability of bacterial species to degrade them and to establish the rerelease of toxic phenols and glucuronic acid, with resulting potential toxicity and/or roles as metabolites.

As an illustration of their utility, 4-methoxyphenyl glucuronide [*p*-methoxyphenyl β-d-glucuronide (**10**)], and the 3- and 4′-glucuronides of resveratrol (**15**) and (**16**) (Scheme 3), which we reported recently,^39^ were tested for susceptibility to hydrolysis by a panel of cell lysates from 9 *Bacteroides* species from the HGM revealing that the glucuronides underwent degradation but that the extent varied between species. Furthermore, it provided evidence that the products may also vary as a function of the bacterial species. Thus, 4-methoxy glucuronide (**10**) was degraded to some extent by the lysate of all 9 bacterial species (Figure 1B), while only the lysate from *B. cellulosilyticus* (Figure 1B, lane 2 and Figure 1C, lane b) produced a product that charred on TLC. The degradation of the resveratrol glucuronides [(**15**) and (**16**)] was most effectively achieved by lysates of different species to those that degraded (**10**); the 3-*O*-resveratrol glucuronide (**15**) also generating slower running products that charred on TLC (Figure 2A, lanes 6 and 9; Figure 2B, lane 9).

The findings are broadly consistent with the varied degradation rates observed for the synthetic model substrate, *p*-nitrophenyl β–d-glucuronide, which have been measured for several *Bacteroides* species^51^ but, extends the findings to naturally occurring glucuronide substrates and reveals differences between them. On the other hand, it will also be interesting to explore the fate of the glucuronide forms of unnatural phenolic compounds, for example, 4-chlorophenol, the phenolic component of clofibrate, a drug used originally to control high cholesterol and triacylglyceride levels in the blood but which was discontinued in 2002 as a consequence of unexplained mortality, despite successfully lowering blood cholesterol levels.^52^ It is interesting to speculate whether the variable degradation of such xenobiotics by gut bacteria may account, at least in part, for their varied efficacy and toxicity.

One interesting property of resveratrol is its ability to isomerize between *cis*- and *trans*-forms, catalyzed by UV light, a feature that we recently showed also applies to the 3-*O*-glucuronide derivative but not readily to the 4′-form.^39^ This observation, which may also help to explain the variable findings regarding the biological activities of resveratrol glucuronide derivatives in the literature, also raises the question of whether the *cis*- or *trans*-forms of resveratrol are equally amenable to glucuronide formation in the host and whether their glucuronides are equally susceptible to hydrolysis by bacterial lyase action. It seems likely, therefore, that exposure of resveratrol to ultraviolet light prior to ingestion, relative rates of glucuronide formation, and their potential hydrolysis by lyases will all need to be understood in greater detail.

Regarding the degradation of the glucuronides by bacterial lysates from human gut bacteria, there was evidence in some cases for the production of, as yet, unidentified products, running with different *R*_f_ values on TLC (Figures 1B,C and 2A,B), and for the 4-methoxyphenyl glucuronide, there was no evidence by HPLC for the release of 4-methoxyphenol (Figure 1D).

Upon addition of 1 mM glucuronic acid to minimal media without glucose [summarized in Figure 4, growth curves and controls shown in Supporting Information (Figure S37)], there was a considerable delay in growth for all nine species and a decrease in overall growth for at least five species; 1, 3, 4, 8, and 9. The remainder, species 2, 5, 6, and 7, had not completed the exponential growth phase by the end of the recording period. The Entner–Doudoroff (ED) pathway, characterized by the key enzyme, 2-keto-3-deoxygluconate-6-phosphate (KDPG) aldolase, is an alternative path by which pyruvate can be generated for the TCA cycle in bacteria, and is known, for example, in *B. caccae*.^53^ It provides a route for glucuronic acid metabolism, which seems to be active among all of the species tested, albeit at the cost of delayed growth and reduced overall growth. In the wider context of the HGM, if this also proves to be the case in vivo, then hydrolysis of glucuronides to release glucuronic acid by *Bacteroides* may provide glucuronic acid as a substrate for bacteria other than *Bacteroides* that are also equipped with the ED pathway, such as *E. coli*,^54^ at least for an initial period. Whether there is any reciprocal effect; these other species perhaps providing nutrients at a later stage of growth for *Bacteroides*, remains a possibility.

The synthetic approach adopted here was also applied to other phenolic compounds. For glucuronides formed with fluorine-containing phenolic moieties, such as 4-fluorophenol, ^19^F NMR offers an attractive route for detection and quantification of degradation since it is essentially free of background signals. Such measurements can be readily adapted for a range of ex vivo or environmental applications in which, ordinarily, very complex background signals would obscure signals from the glucuronide or products of hydrolysis, whatever conventional detection system—HPLC, mass spectrometry or ^1^H/^13^C NMR spectroscopy—were being employed.

Direct toxic effects or their potential as metabolites are not, however, the only possible routes by which phenols and their glucuronides can influence bacterial growth. There is also the possibility that they may interfere with bacterial sensing mechanisms. Many bacterial and archaeal species employ methyl-accepting chemotaxis proteins (MCPs) to detect their immediate chemical environment.^55,56^ For example, *E. coli* has four MCPs through which it can respond to the signal either as an attractant (Tar) or repellent (Tyn, Try, and Tsr).^27,57^ Additionally, the principle that phenolic compounds can inhibit the quorum sensing apparatus of bacteria has been established, and even though the target species are not usually classified as members of the healthy HGM, they are, nevertheless, capable of opportunistic infections during periods of dysbiosis (e.g. *Chromobacterium violaceum*, *Pseudomonas aeuruginosa,* and *Serratia marcescens)*.^26,56^ Quorum sensing systems are also employed by common gut bacteria including *Bacteroides*(57) and *C. difficile,*(59,60) and resveratrol inhibits quorum sensing by altering biofilm formation in *P. aeruginosa* PAO1.^58^ The potential release of GlcA, whose open-chain form possesses an aldehyde group with participation in Schiff’s base formation with nitrogen-containing nucleophiles,^61^ offers a further route by which the products of the hydrolysis of glucuronides by the bacterial populations of both the healthy and dysbiotic human gut microbiome could act.

The compounds reported here, together with those reported earlier,^38,39^ comprise a toolkit with which these interactions can be investigated in the future. The results further highlight the differential degradation of glucuronides, a property that has been noted in relation to the reactivation of the glucuronides of xenobiotics by β-glucuronidases and has led to glucuronidases becoming the target of inhibition.^62^ These degradative pathways may also have potential in relation to dietary glucuronides.

The use of model glucuronides as substrates was illustrated in studies of GUS activity using cell lysates of 9 species of HGM (*Bacteroidetes*), revealing distinct degradation outcomes on both these naturally occurring glucuronides and the glucuronide of 4-fluorophenol, whose degradation was monitored by ^19^F NMR, providing background-free and sensitive detection of β-glucuronidase activity. This may provide a convenient, essentially background-free method for monitoring β-glucuronidase activity in samples, where the signals arising from the starting material and products of model glucuronides are obscured by the complexity of background signals when using other methods.

## Supporting Information Available

Experimental procedures, bacterial lysate experiments, general experimental procedures, NMR spectra for compounds (**5**), (**6**), (**7**), (**8**), (**9**), (**10**), (**18**), and (**19**), and bacterial growth curves (PDF)
