# Supplementary material for: Synthetic β-d-Glucuronides: Substrates for Exploring Glucuronide Degradation by Human Gut Bacteria
Source: ACS Omega. 2024 Dec 20;10(1):1419–28. doi: 10.1021/acsomega.4c09036 (PMC11740244; doi:10.1021/acsomega.4c09036)
Supplement: Supplementary file 1 — ao4c09036_si_001.pdf [file ao4c09036_si_001.pdf]

Supplementary Information

Synthetic β–D-glucuronides; substrates for exploring glucuronide degradation by human gut bacteria

Aleksandra Gorecka[1,6], Heidi Schacht[2], Megan K Fraser[1], Aleksandra Teriosina[3], James A London[1,7], Igor L Barsukov[2], Andrew K Powell[4], Alan Cartmell[5], Andrew V Stachulski[1*] and Edwin A Yates[2*]

[1]*Department of Chemistry, University of Liverpool, Liverpool L69 7ZD, U.K.*

[2]*Department of Biochemistry, Cell and Systems Biology, ISMIB, Crown St., University of Liverpool, Liverpool L69 7ZB, U.K.*

[3]*School of Biological Sciences, University of Liverpool, Crown Street Liverpool, L69 7ZB, UK.*

[4]*School of Pharmacy and Biomolecular Sciences, Liverpool John Moores University, Byrom Street, Liverpool, L3 3AF, UK.*

[5]*Department of Biology, University of York, Heslington, York, YO10 5DD, UK.*

[6]*Current address: School of Chemistry, Cardiff University, Park Place, Cardiff, CF10 3AT UK.*

[7]*Current address: Department of Biochemistry, Sanger Building, University of Cambridge, Old Addenbrookes Site, Tennis Court Road, Cambridge, CB2 1GA UK.*

**joint corresponding authors*

Contents **Page**

1. Experimental procedures S1
2. Example 1H and 13C NMR spectra; 2-methoxyphenyl-β-D-glucopyranosyluronate (**8**) S7
3. Bacterial lysate experiments S8
4. General experimental procedures S15
5. NMR spectra for compounds (**5**), (**6**), (**7**), (**8**), (**9**), (**10**), (**18**) and (**19**) S16
6. Bacterial Growth Curves S25

---

1. Experimental procedures.

1.1 Synthesis of methoxyphenyl β-D-glucuronides via anomeric tetraester coupling.

General glucuronidation method: Methyl-1,2,3,4-tetra-O-acetyl-β-D-glucopyranosyluronate (**1**) and the phenol (**2**), (**3**) or (**4**) (1 eq. of each) were dissolved in anhydrous dichloromethane (4mL per mmol), then stirred for 2 h under N_2 [at 20°C] with 4Å molecular sieves. Trimethylsilyl trifluoromethanesulfonate (1

eq.) was added to the reaction mixture, which was stirred for a further 3.5 h. The reaction mixture was quenched with saturated aqueous sodium bicarbonate (20 mL per mmol) and diluted with ethyl acetate (20 mL per mmol). After separation, the organic layer was washed with water followed by brine, then dried with anhydrous $MgSO_4$. Following filtration and evaporation under reduced pressure, the crude product was purified by column chromatography using a gradient of 30-50% ethyl acetate in hexane to afford the respective glucuronide esters detailed below.

Methyl-1-(2-methoxyphenyl)-2,3,4-tri-O-acetyl-β-D-glucopyranosyluronate (**5**):- From (**2**) (0.40 g, 3.19 mmol) was obtained (**5**) (0.77 mmol, 24% yield) after recrystallisation from EtOAc : ^{1}H NMR (400 MHz, acetone-d_6) 2.00-2.05 (3s, 9H, 3xOAc), 3.71 (s, 3H, CO_2Me), 3.83 (s, 3H, $ArOCH_3$), 4.50 (d, J = 10.0 Hz, 1 H, 5-H), 5.20-5.25 (m, 2H, 2-H + 4-H), 5.36 (d, J = 8.0 Hz, 1 H, 1-H), 5.44 (t, J = 9.6 Hz, 1 H, 3-H), 6.89 (dt, J = 8.0 and 1.8 Hz, 1H, ArH), 7.02-7.07 (m, 2H, ArH) and 7.17 (dd, J = 8.0 and 1.7 Hz, 1H, ArH); ^{13}C NMR (100 MHz, acetone-d_6) 19.5, 19.6, 19.7, 52.0, 55.5, 69.5, 71.0, 71.6, 72.0, 100.1, 113.0, 119.7, 120.7, 124.5, 146.2, 150.8, 167.1, 168.7, 169.0 and 169.3; Found: C, 54.2; H, 5.5; m/z, 463.1216 (MNa^+, ESI +ve mode); $C_{20}H_{24}0_{11}$ requires C, 54.5; H, 5.5%; $C_{20}H_{24}0_{11}Na$ requires m/z, 463.1216.

General hydrolysis method: The appropriate ester (**5**), (**6**) or (**7**) dissolved in MeOH (15 mL per mmol) was treated with a solution of anhydrous Na_2CO_3 (3 eq.) in water (4 mL per mmol) added over 1 minute at 0°C. The resulting mixture was allowed to regain ambient temperature and further H_2O added as necessary to give a clear solution. When reaction was complete by TLC, Amberlite IR-120 (H^+) was added to give a pH of 6, then the solution was filtered, evaporated to dryness and azeotroped with EtOH (3 portions) to give the product. Further purification if necessary was achieved using gel permeation chromatography with Bio-gel P2 to give the final products (**8**), (**9**) and (**10**).

2-Methoxyphenyl-β-D-glucopyranosyluronate, sodium salt (**8**):- On a 0.5 mmol scale from (**5**), obtained (**8**) (0.085 g, 53%). Following gel permeation chromatography with Bio-gel P2 to obtain pure product: ^{1}H NMR (600 MHz, D_2O) δ 3.53-3.57 (m, 3 H, H-2 + H-3 + H-4), 3.78 (d, J = 9.2 Hz, 1 H, H-5), 3.81 (s, 3H, ArOMe), 5.06 (d, J = 7.6 Hz, 1 H, H-1), 6.95 (m, 1H, ArH), 7.06 (m, 2 H, ArH) and 7.12 (dd, J = 8.0 and 2.0 Hz, 1H, ArH); ^{13}C (150 MHz, D_2O) δ 181.5, C-6 ($ArCH_3$), 148.7, 145.4, 123.9, 121.6, 116.4, 113.0 (6C aromatics), 100.4, C-1, 76.3, C-5, 75.3, C-3, 72.7, H-2, 71.7, H-4, 55.8.

Methyl-1-(3-methoxyphenyl)-2,3,4-tri-O-acetyl-β-D-glucopyranosyluronate (**6**):- From 3-methoxyphenol **(3)** (0.33 mL, 3 mmol) was obtained (**6**) (0.86 mmol, 27%): ^{1}H NMR (400 MHz, DMSO-d_6) δ_H 2.00-2.02 (3s, 9H, OAc), 3.64 (s, 3H, ArOMe), 3.74 (s, 3H, CO_2Me), 4.72 (d, 1H, J = 10 Hz, 5-H), 5.04-5.10 (m, 2H, 2-H + 4-H), 5.46 (d, 1H, J = 9.6 Hz, 3-H), 5.67 (d, 1H, J = 8.0 Hz, 1-H), 6.55 (t, 1 H, J = 2 Hz, Ar 2-H), 6.59 and 6.67 (2dd, J = 8 Hz and 2 Hz, Ar 4-H and Ar 6-H) and 7.24 (t, J = 8 Hz, Ar 5-H); ^{13}C NMR (100 MHz, $CDCl_3$) δ_H 20.5, 20.6, 53.0, 55.4, 69.1, 71.0, 71.9, 72.7, 99.1, 103.6, 108.9, 109.0. 130.0, 157.8, 160.8, 166.8, 169.2 169.3 and 170.1; only two distinct CH_3CO; Found: m/z, 463.1219 (MNa^+, ESI +ve mode); $C_{20}H_{24}0_{11}Na$ requires m/z, 463.1216.

3-Methoxyphenyl-β-D-glucopyranosyluronate, sodium salt (**9**):**-** On a 0.5 mmolar scale, from (**6**) was obtained (**9**) (0.11 g, 68%): ^{1}H NMR (400 MHz, D_2O) δ_H 3.81 (d, 1H, J = 10 Hz, 5-H), 3.50-3.60 (m, 3H, 2-H, 3-H and 4-H), 3.75 (s, 3H, ArOMe), 5.04 (d, J = 6.4 Hz, 1-H), 6.67-6.76 (m, 3H, Ar 2-H, Ar 4-H and Ar 6-H) and 7.25 (t, 1H, J = 8.4 Hz, Ar 5-H); δ_C 55.5, 71.7, 72.8, 75.3, 76.2, 100.1, 103.1, 108.9, 109.1, 130.6, 157.8 and 160.1; one aryl C not seen; Found: (ESI +ve mode) m/z, 323.0744; $C_{13}H_{16}O_8Na$ (MH^+ for Na salt) requires m/z, 323.0743.

Methyl-1-(4-methoxyphenyl)-2,3,4-tri-O-acetyl-β-D-glucopyranosyluronate (**7**):- From 4-methoxyphenol (**4**) (0.38 g, 3.1 mmol) was obtained compound (**7**) (0.53 g, 1.21 mmol, 38%)**:** ^{1}H NMR (400 MHz, DMSO-d_6) δ 2.01-2.04 (3s, 9H, OAc), 3.65 (s, 3H, OMe), 3.73 (s, 3H, OMe), 4.66 (d, 1H, J = 10.0 Hz, 5-H), 5.03-5.08 (m, 2 H, 2-H + 4-H), 5.46 (t, 1 H, J= 9.6 Hz, 3-H), 5.50 (d, J = 8.0 Hz, 1-H) and 6.88-6.96 (2dd, J = 6.8 and 2.4 Hz, 4H, ArH); ^{13}C NMR (100 MHz, DMSO-d_6) δ_C 20.7, 20.8 (x2), 53.0, 55.9, 69.5, 71.1, 71.4, 71.6, 98.7, 115.2, 118.4, 150.7, 155.7, 167.6, 169.5, 169.8 and 170.0.

4-Methoxyphenyl-β-D-glucopyranosyluronate, sodium salt (**10**) :- From ester (**7**) (0.213 g, 0.5 mmol) was obtained compound (**10**) (0.11 g, 0.35 mmol**,** 71%) after purification using gel permeation chromatography with Bio-gel P2: ^{1}H NMR (400 MHz, D_2O) δ_H 3.50-3.53 (m, 3H, 2-H + 3-H + 4=H), 3.73 (s, 3H, CH_3O), 3.75 (d, J = 9.1 Hz, 1 H, 5-H), 4.91 (d, J = 7.3 Hz, 1 H, 1-H), 6.89 (d, J = 7.2 Hz, 2H, ArH) and 7.04 (d, J = 7.2 Hz, 2H, ArH); ^{13}C (100 MHz, D_2O) δ_C 55.8, 71.7, 72.8, 75.3, 76.2, 101.2, 115.0, 118.4, 151.0, 154.7 and 175.4; Found: (ESI –ve mode) m/z, 299.0773; $C_{13}H_{15}O_8$ ($[M-H]^+$ for CO_2^-) requires m/z, 299.0767.

***1.2** Synthesis of methoxyphenyl β-D-glucuronides via the trichloroacetimidate method.*

Methyl 2,3,4-tri-O-acetyl-α, β-D-glucopyranosyluronate (**13**):- This was prepared from tetraester (**1**) using N-Me piperazine as recently described [39]. On a 4 mmolar scale, obtained hemiacetal (**13**) (1.18 g, 88%) as a colourless oil, α:β ~3:1: ^{1}H NMR (400 MHz, $CDCl_3$) δ 2.05-2.11 (6s, 9H, OAc α/β), 3.77, 3.78 (2s, 3H, CO_2Me α/β), 4.13 (d, J = 9.6 Hz, 1H, 5-Hβ) 4.61 (d, J = 10.0 Hz, 5-Hα), 4.81 (d, J = 7.6 Hz, 1-Hβ), 4.95 (dd, J = 10.0 and 2.0 Hz, 2-Hα), 5.05 (dd, J = 9.2 and 1.2 Hz, 2-Hβ), 5.19-5.35 (m, 4-Hα + 3-Hα + 3-Hβ), 5.46 (m, 1-Hα) and 5.60(t, J = 9.7 Hz, 4-Hβ); ^{13}C NMR (100 MHz, $CDCl_3$) 170.72, 170.10, 170.00, 169.65, 169.52, 168.34, 167.50, 95.58, 90.28, 72.95, 72.65, 71.39, 70.71, 69.49, 69.04, 68.09, 53.04, 52.91, 21.05, 20.88, 20.68, 20.60, 20.54, 20.50.

Methyl 2,3,4-Tri-O-acetyl-1-O-(trichloroacetimidoyl)-α-D-glucopyranosyluronate (14):- This was obtained from hemiacetal (**13**) as described previously, [39] using $Cl_3C.CN$ and K_2CO_3 in CH_2Cl_2 . On a 3 mmolar scale obtained (**14**) (1.28 g, 89%) which was readily crystallised from EtOAc-hexane: ^{1}H NMR (400 MHz, $CDCl_3$) δ 2.01 (s, 3H, OAc), 2.08 (2s, 6H, OAc), 3.76 (s, 3H, CO_2Me), 4.52 (d, J = 10.2,1H, 5-H), 5.17 (dd, J_1 = 10.01, J_2 = 3.6, 1H, 2-H), 5.29 (t, J = 10, 1H, 4-H), 5.64 (t, J = 9.9, 1H, 3-H), 6.66 (d, J = 3.6, 1H, 1-H) and 8.76 (s, 1H, NH).

Methyl-1-(2-methoxyphenyl)-2,3,4-tri-O-acetyl-β-D-glucopyranosyluronate (**5**):- Methyl 2,3,4-Tri-O-acetyl-1-O-(trichloroacetimidoyl)-α-D-glucopyranuronate (**14**) (0.22 g, 0.46 mmol) and 2-methoxyphenol (**2**) (0.04 ml, 0.32 mmol) were dissolved in anhydrous dichloromethane (16.5 ml) and stirred with 4 Å molecular sieves for 30 min. The reacting mixture was cooled down to -20°C and $BF_3{\cdot}OEt_2$ (0.01 ml) was added dropwise. After regaining ambient temperature, the solution was left to stir for a further 4 h. The mixture was quenched with saturated sodium bicarbonate solution (20 ml) and then extracted with dichloromethane (3 x 10 ml). Combined organic layers were washed with water (20 ml), brine (20 ml) and then dried over magnesium sulphate. Solvents were evaporated under reduced pressure. Product was purified using flash chromatography, eluting with hexane/ethyl acetate (2:1) solution to obtain (**5**) (0.082 g, 0.186 mmol, 58%): ^{1}H NMR as for (**5**) above.

2-Methoxyphenyl-β-D-glucopyranosyluronate, sodium salt (**8**):- Methyl-1-(2-methoxyphenol)-2,3,4-tri-O-acetyl-β-D-glucopyranuronate (**5**) (80 mg, 0.248 mmoles) was suspended in methanol (3 ml) and stirred at 0°C while a solution of sodium carbonate (0.143 g, 1.35 mmoles) in water (5 ml) was added over 1 min. The mixture was allowed to regain ambient temperature, then after an additional 2 h, water

(1 ml) was added to afford a clear solution. After 3h, Amberlite IR-120 (H+) resin was added with stirring until a pH of 6.0 was obtained. The resin was filtered, then the filtrate evaporated to dryness to provide (**8**) (0.231 mmoles, 93%): ^{1}H and ^{13}C NMR data as for (**8**) above.

Methyl-1-(4-fluorophenyl)-2,3,4-tri-O-acetyl-β-D-glucopyranosyluronate (18):-
This compound was prepared from 4-fluorophenol **17** using either the anomeric tetraester or trichloroacetimidate methods (v. s.) in 22% and 69% yields, respectively. Compound **18**: 1H NMR (400 MHz, DMSO-d_6) 2.00, 2.01 and 2.04 (9 H, 3s, 3xCH_3CO), 3.64 (3H, s, CH_3O), 4.69 (1 H, d, J = 10.0 Hz, 5-H), 5.04-5.11 (m, 2 H, 2-H + 4-H), 5.46 (t, J = 9.6 Hz, 1 H, 3-H), 5.60 (d, J = 8.0 Hz, 1 H, 1-H), 7.03 (m, 2 H, ArH) and 7.15 (t, J = 8.0 Hz, 2 H, ArH); 13C NMR (100 MHz, DMSO-d_6) 20.7, 20.8 (x2), 53.1, 69.4, 70.9, 71.4, 71.5, 98.1, 116.7 (d, J = 23 Hz), 118.7 (d, J = 8 Hz), 153.0 (d, J = 2 Hz), 158.4 (d, J = 237 Hz), 167.5, 169.5, 169.8 and 170.0; 19F NMR: -120.6. Found: C, 53.36; H, 4.9; m/z, 451.1016 (MNa^+, ESI +ve mode); $C_{19}H_{21}FO_{10}$ requires C, 53.3; H, 4.9%; $C_{19}H_{21}F0_{10}$.Na requires m/z, 451.1016.

4-Fluorophenyl-β-D-glucopyranosyluronate, sodium salt (19):-
Ester **18** (0.210 g, 0.49 mmol) was stirred in methanol (5 mL) at 0°C then 0.5 M NaOH (1.0 mL) was added over 12 min. The solution was allowed to regain 20°C over 1 h, then further 0.5 M NaOH (0.04 mL) was added. After stirring for another 0.25 h, the solution was evaporated to dryness then azeotroped with ethanol (3x 5 ML) to afford **19 as** a white solid (0.160 g), which was sufficiently pure for assays. ^{1}H NMR (700 MHz, D_2O) δ_H 3.53-3.49 (3 H, m, 2-H, 3-H, 4-H), 3.77 (1 H, d, J=8.9 Hz, 5-H), 4.95 (1 H, d, J=8.8 Hz, 1-H), 7.017-7.068 (4 H, m, ArH); (175 MHz, D_2O) δ_C 71.6, 72.8, 75.5, 76.3, 101.0, 116.1 (d, $^{2}J_{C\text{-}F}$ 18 Hz), 118.5, 152.8, 158.6 (d, $^{1}J_{C\text{-}F}$ 231 Hz) and 176.0; δ_F (564 MHz, D_2O) -125.1 (s, 1F) relative to 4-fluorphenol in D_2O (as external standard = -125.1 (s)). Found: m/z, 287.0564 (ESI -ve mode); $C_{12}H_{12}F0_7$ requires m/z, 287.0567.

1.3 *Hydrolysis of glucuronide model substrates by Bacteroides species from the human gut microbiota.*
1.3.1 Bacterial culture and lysate preparation.
The 9 bacterial samples were grown anaerobically in BHI media supplemented with haematin (120 µg/ml) to mid-late log phase (OD *ca*. 0.8), 5 mL of culture medium was removed, spun and washed (x 2) with PBS (5 ml), before a final resuspension in 1 ml of PBS. Cells were sonicated (Sonics Vibracell, with ultrasound probe for 2 x 15s at 25% power). Twenty µL of lysed bacterial supernatant was added to the chosen glucuronide (5 µL, 1 mg/ml) in PBS and incubated (37°C).

Growth curves were conducted over 48h reading every 3 mins, in a 96-well plate. Experiments measuring the effect of phenolic compounds, their respective glucuronides (1 mM) were added to BHI media cultures, while for experiments to assess the effect of glucuronic acid (1 mM), the compounds were added to the cultures in minimal media in the absence of glucose.

1.3.2 Analysis of the extent of glucuronide hydrolysis by TLC.

The incubated glucuronide was subjected to TLC (silica, Merck) run in EtOAc/MeOH 1/1, 1 ascent, or methanol (1 ascent) as indicated and compounds revealed by charring in 10% concentrated H_2SO_4 in EtOH. For comparative purposes, equal loading of control and experimental lanes (2 µg per spot).

1.3.3 Analysis of products by HPAEC.

Samples were boiled, centrifuged and diluted 1:100 into 300 µl of which 100 µl was loaded onto the column. Samples were run on a thermofisher (Dionex) ICS-6000 ion chromatography system and detected using an electrochemical detector via pulsed amperometric detection, using the “Gold, Carbo, Quad” waveform. For separation a PA-200 Carbopac anion exchange column (3 x250 mm), with a preceding PA-200 guard column (3 x 50 mm), was used. Samples were eluted off the column with an initial isocratic flow of 0.1 M NaOH for 10 mins; 10 min to 40 min a linear gradient to 50 % with the eluent 0.1 M NaOH with 1 M NaOAc was used; a wash step of 100 % 0.1 M NaOH with 1 M NaOAc was run (10 mins) followed by a wash step of 0.5 M NaOH (10 mins) before a 10 min equilibration back into 0.1 M NaOH; total run time was 70 mins using a flow rate of 0.25 ml/min.

1.3.4 NMR Spectroscopy.

Samples were prepared by dissolving samples in 700 µL of D_2O at 343 K and spectra shown in the paper were recorded using a 600 MHz Bruker Avance II+ spectrometer equipped with a TCI cryoprobe. 1H and 13C data provided in the SI were recorded at 400 (1H)/100 (13C) MHz, 600/150 MHz for 13C) or 700 MHz/175 MHz for 13C). Chemical shift values (^1H) are quoted relative to DSS (0 ppm). ^{19}F NMR NMR spectra were recorded on a Bruker Ascend 600 MHz instrument, at 299K, recording 2048 scans. Spectra were processed using Bruker TopSpin and integration was performed with Bruker TopSpin or INFOS spectrum fitting software.

2 **Figure S1**. ^{1}H (upper) and ^{13}C (lower) NMR spectra of 2-methoxyphenyl-β-D-glucopyranosyluronate (8) in D_2O. The acetate signal is from sodium acetate.

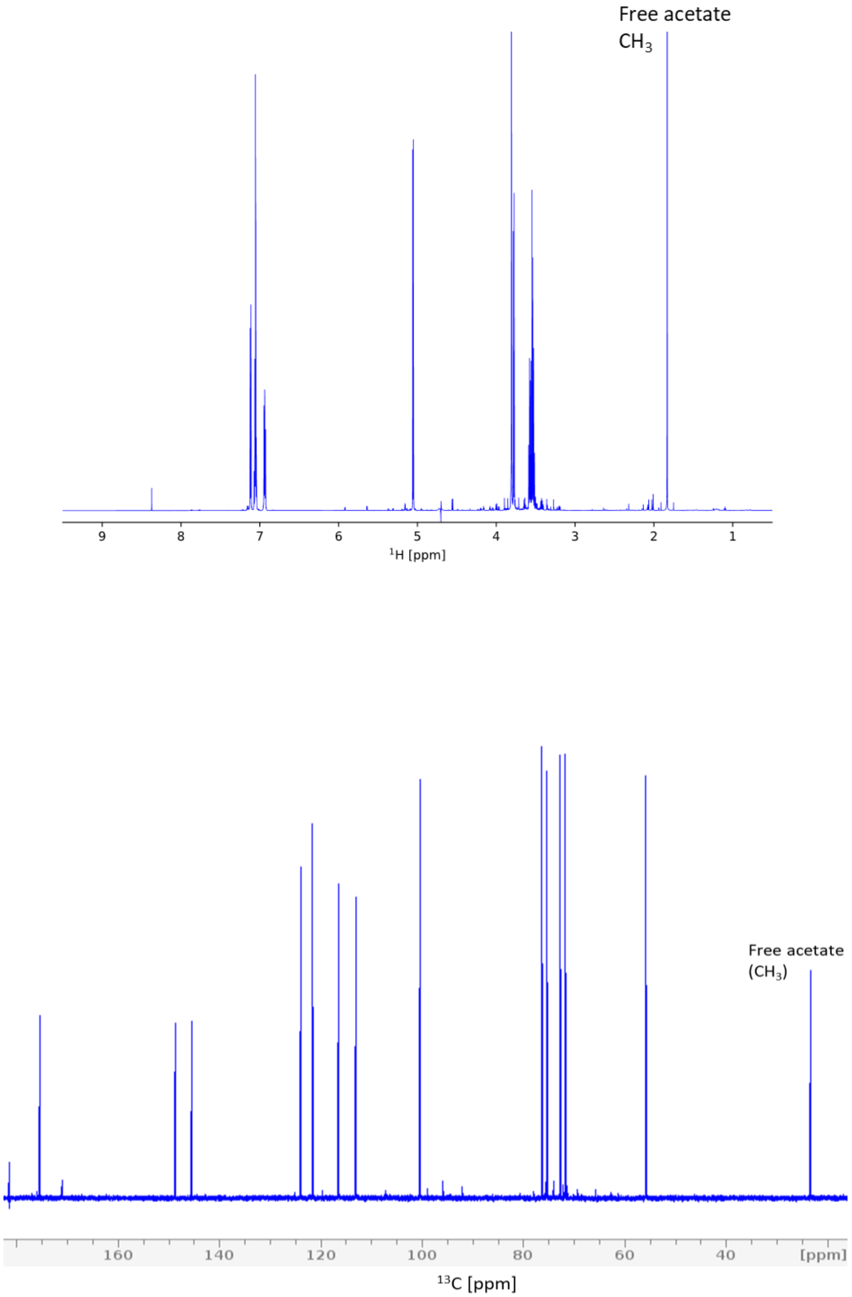

3 Bacterial cell lysate experiments.

Figure S2. Fast-running bands on TLC all derive ultimately from glucuronides.

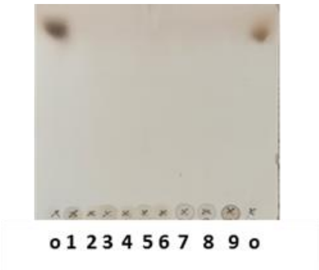

Thin layer chromatogram (methanol, 1 ascent) showing lysate only control. Lane o (left); resveratrol 3-O-β-D-glucuronide alone; lanes 1-9 correspond to the 9 bacterial lysates; lane o (right); resveratrol 4'-O-β-D-glucuronide.

Figure S3. NMR analysis of un-digested resveratrol glucuronide and additional products.

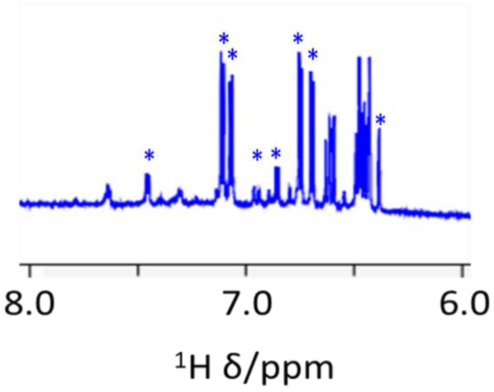

^{1}H NMR (detail) showed that intact resveratrol 3-O-β-D-glucuronide (persisting in the E-isomer form (marked by *)) as well as additional products, not consistent with free resveratrol, were released by bacterial cell lysates acting on resveratrol 3-O-β-D-glucuronide. See also 3.3 below.

Figure S4. TLC comparison 4-methoxyphenol and its glucuronide.

TLC showed that the faster running band on TLC (see **Figure 1B** lane 2 and **Figure 1C** lane b in main text), while consistent with released 4-methoxyphenol (below), further investigation by HPLC (**Figure 1D** in main text) revealed that this product is not liberated 4-methoxyphenol. This was confirmed by subsequent ^{1}H NMR analysis (see **Figure S3.9**).

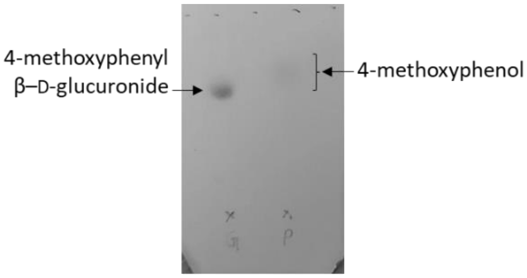

Solvent ethyl acetate/methanol (1/1, v/v 1 ascent). Charring, 10%, v/v conc. sulfuric acid/ethanol.

TLC of lysate of bacteria in lane 2 with 4-methoxyphenyl glucuronide (left) and the putative released phenolic moiety, 4-methoxyphenol (right).

Figure S5. TLC of resveratrol and its 3-O-glucuronide.

The slower running band on TLC (see **Figure 2A**, lanes 6 and 9, and **Figure 2B**, lane 9; methanol, 1 ascent) is not consistent with resveratrol being released by lyase action and implies formation of an additional metabolite, as outlined above in 3.1B.

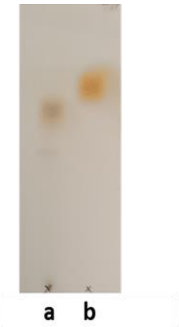

Thin layer chromatogram (methanol, 1 ascent) showing: lane **a**, resveratrol 3-O-β-D-glucuronide alone; **b** resveratrol alone.

Figure S6. SDS-PAGE gel analysis of bacterial lysates 1-9.

Molecular weight markers are in lanes M with their weights shown alongside.

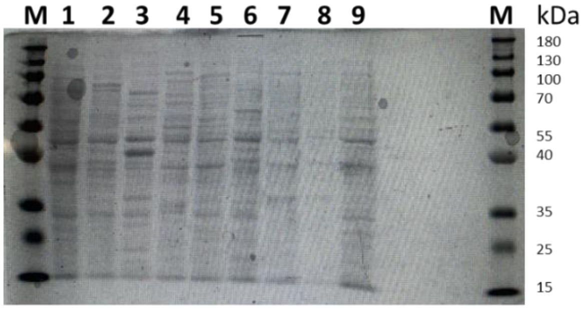

SDS PAGE gel of bacterial lysates 1-9.

The human gut microbiome possesses over 3000 β-glucuronidases comprising 279 unique enzymes in 6 structural groups[51]; those from *Bacteroides* are typically homodimers from around 70 kDa, and include GUS-1 and GUS-3 from *B. uniformis*[34]. The substrate specificity is known to vary considerably between enzymes[64].

64. B.D. Wallace, A.B. Roberts, R.M. Pollet *et al.*, Chem.& Biol., (2015) **22**, 1238-1249.

Figure S7. HPLC traces of glucuronide derivatives.

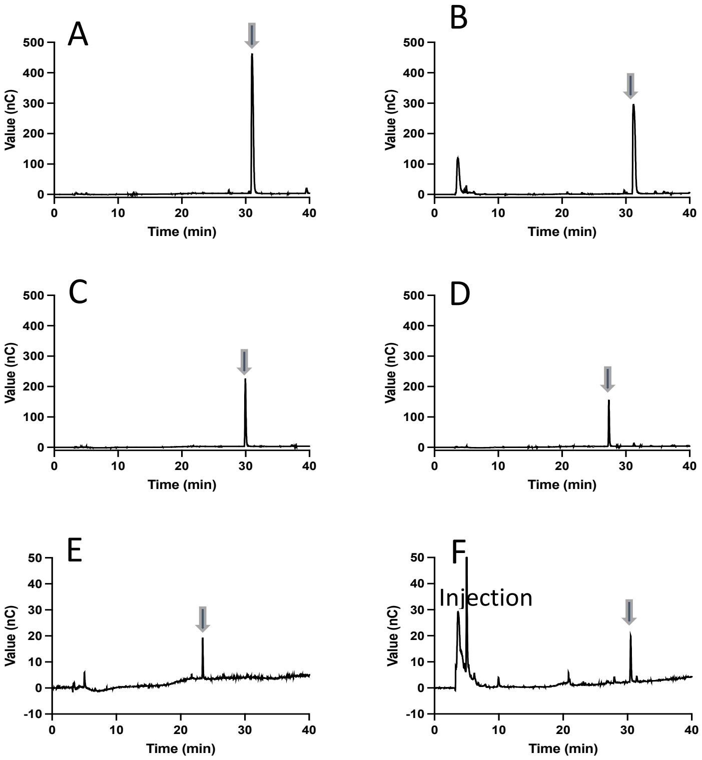

HPAEC traces (electrochemical detection) of: A. -methylphenyl-β-D-glucuronide[38] B. 2-methoxylphenyl-β-D-glucuronide. C. 3-methoxphenyl- β-D-glucuronide D. 4-methoxylphenyl-β-D-glucuronide E. Resveratrol 3- β-D-glucuronide[39] F. resveratrol 4'- β-D-glucuronide[39]. Products marked by an arrow.

Figure S8. ^{19}F NMR detection of 4-fluorophenyl glucuronide (**19**) and its hydrolysis.

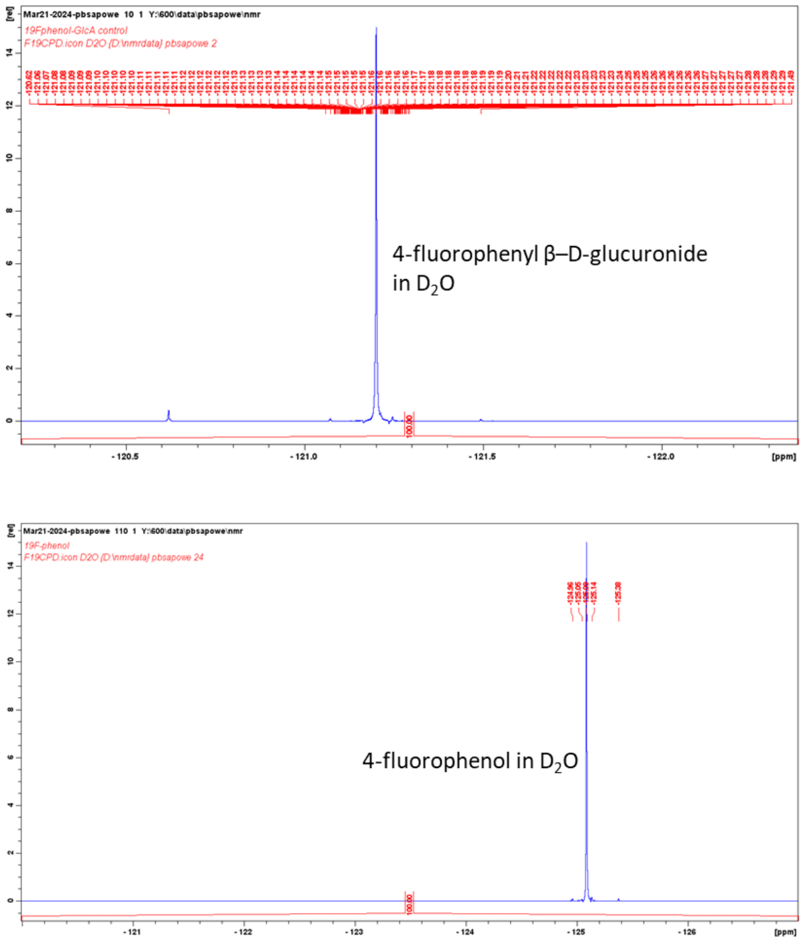

^{19}F NMR spectra of (upper) 4-fluorophenyl β–D-glucuronide and (lower) 4-fluorophenol in D_2O.

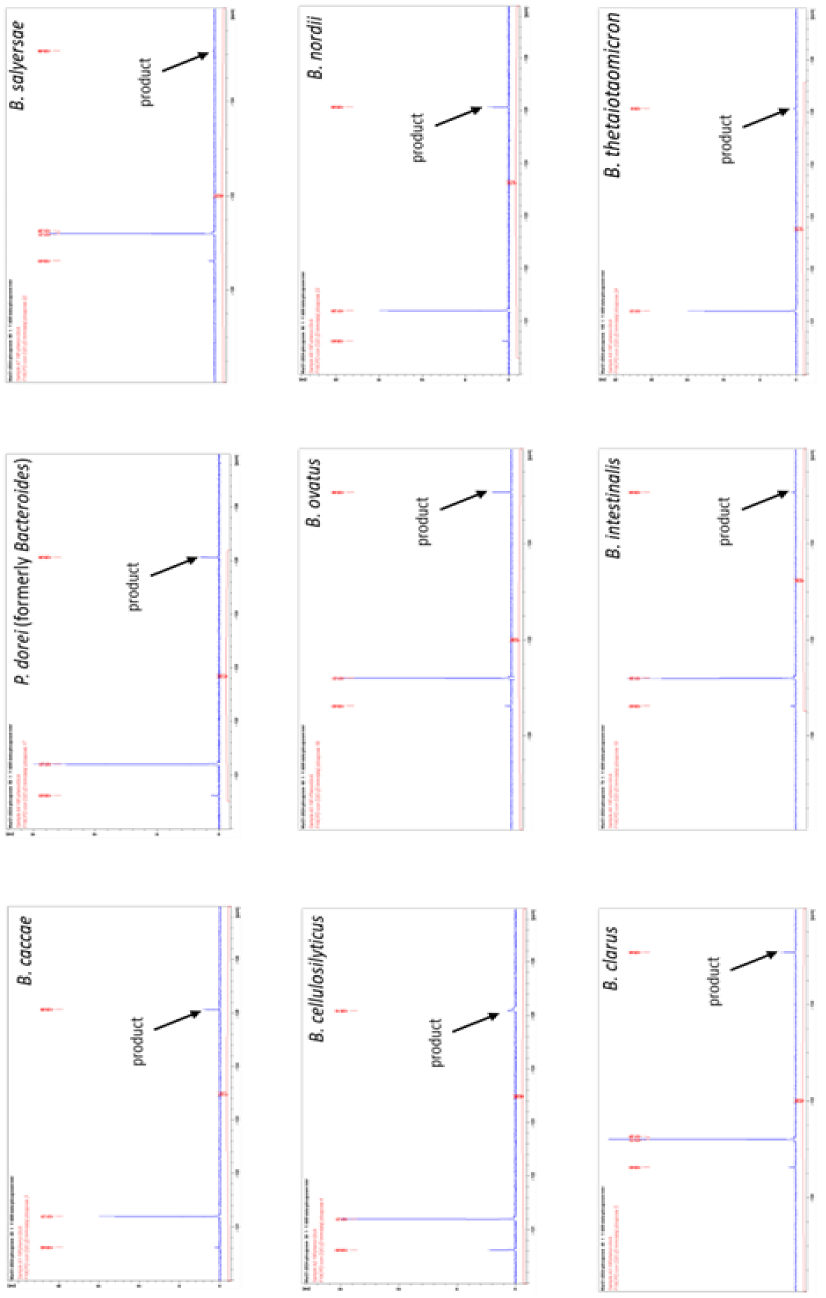

Figure S9. ^{19}F NMR spectra of bacterial lysate digests of 4-fluorophenyl β-D-glucuronide. The 4-fluorophenol product is indicated by an arrow.

Figure S10. ^{1}H NMR analysis shows that free GlcA is not detectable (H-1 signal indicated) in bacterial lysates of 4-fluorophenyl glucuronide with the possible exception of *P. dorei* (product signal circled).

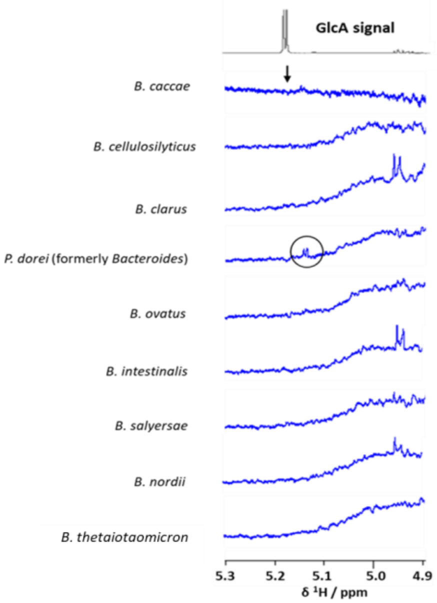

Figure S11. ^{1}H NMR analysis shows that the products of bacterial lysate action vary between bacterial species.

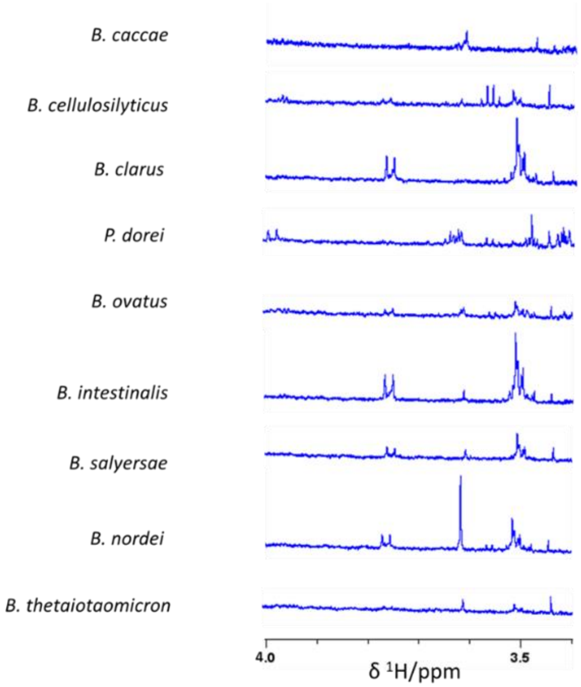

Figure S12. ^{1}H NMR analysis of the lysates of 4-methoxy β-D-glucuronide showing: **A.** No free glucuronic acid remains in any of the lysates. **B.** There is little evidence for free 4-methoxyphenyl glucuronide, with the exception of B*. intestinalis*, for which minor signals are evident. **C**. Differences in signals exist in the aliphatic region of the ^{1}H NMR spectra of the bacterial lysates indicating subtly different products between bacterial species.

A

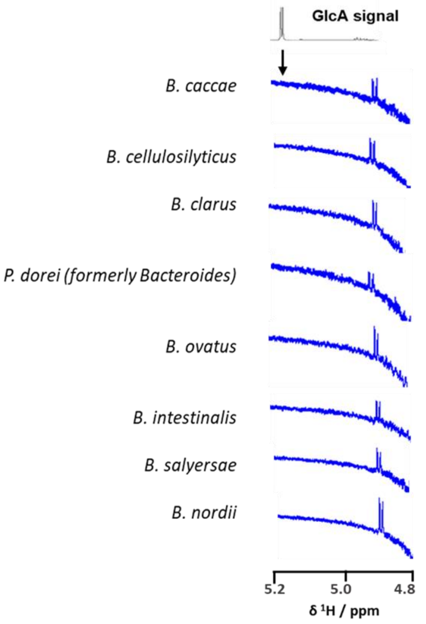

No free glucuronic acid is detectable in the bacterial lysates of 4-methoxyphenyl glucuronide. Detail of ^{1}H NMR spectra.

B

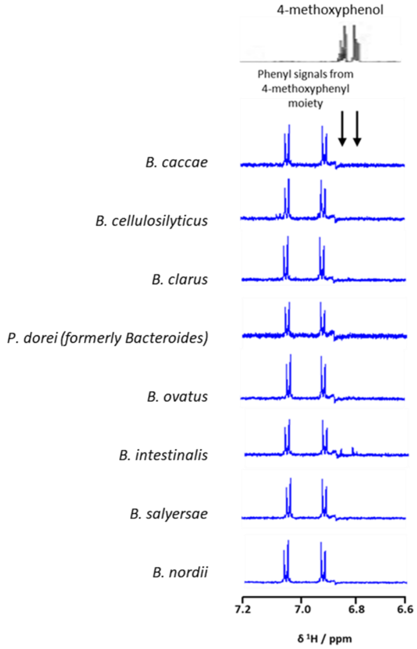

There is little evidence of the release of free 4-methoxyphenol in any of the bacterial lysates, with the exception of *B. intestinalis* which shows small signals from the free phenol. Detail of ^{1}H NMR spectra.

C

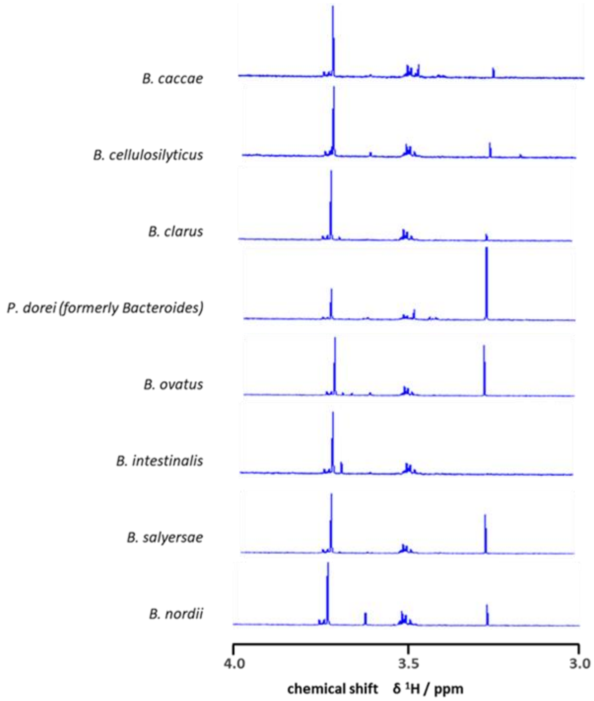

Differences exist in the 3-4 pm region of the ^1H NMR spectra of the bacterial lysates of 4-methoxy glucuronide suggesting distinct products. This region typically indicates aliphatic groups of , for example, the –CH_2OH, -CH_2O-, -CH_2N- types.

4. **General Experimental Procedures.**

Organic extracts were finally washed with saturated brine and dried over anhydrous Na_2SO_4 prior to rotary evaporation at <30°C. Moisture sensitive reactions were carried out in anhydrous organic solvents (purchased from Sigma-Aldrich) under a N_2 or Ar atmosphere. Reactions were monitored by analytical thin-layer chromatography using Merck Kieselgel 60 F_{254} silica plates, and were viewed under UV or by staining with $KMnO_4$ or iodine. Preparative flash column chromatography was performed on either VWR Prolabo silica gel or Sigma-Aldrich silica gel (particle size 40-63 Å). Melting points were recorded using a Bibby-Sterlin Stuart SMP3 melting point apparatus and are uncorrected. Mass spectra were obtained in either electrospray mode (ES) with a Micromass LCT or chemical ionization (CI) mode with a Micromass Trio 1000 using ammonia. Elemental analyses were performed by Mrs. Jean Ellis, University of Liverpool. ^1H and ^{13}C NMR spectra were obtained using Bruker Avance or Bruker DPX 400 (Dept. Chemistry), and Bruker Avance 600 MHz or Bruker Avance II+ 800 MHz instruments (Dept. Biochemistry) the 400, 600 or 800 MHz respectively for 1H spectra and 100, 150 and 200 MHz for 13C spectra. Chemical shifts are reported in ppm (δ) relative to Me_4Si. Coupling constants (*J*) are reported in Hz.

5. NMR spectra for compounds 5, 6, 7, 8, 9, 10 a18

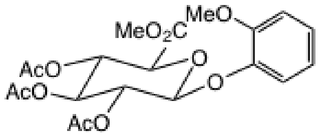

Figure S13. Compound 5

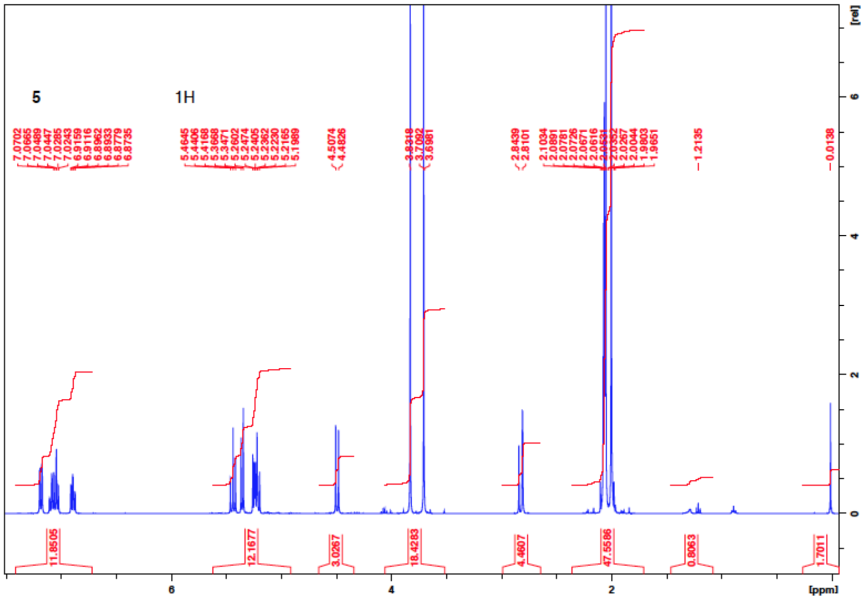

Figure S14.

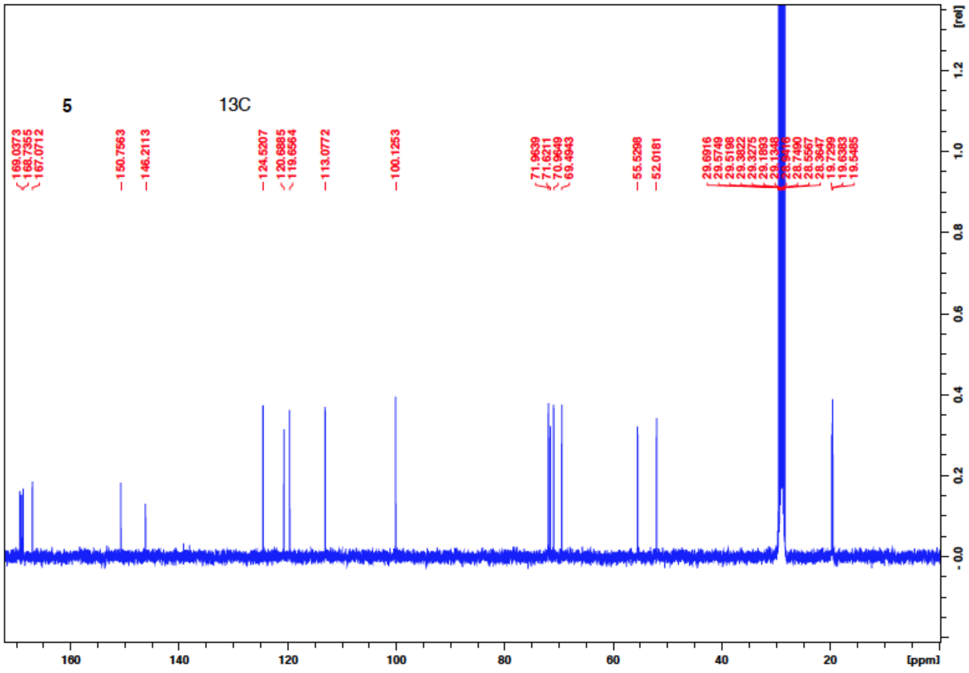

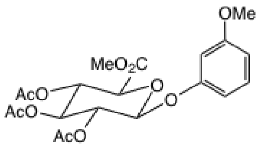

Figure S15. **Compound 6**

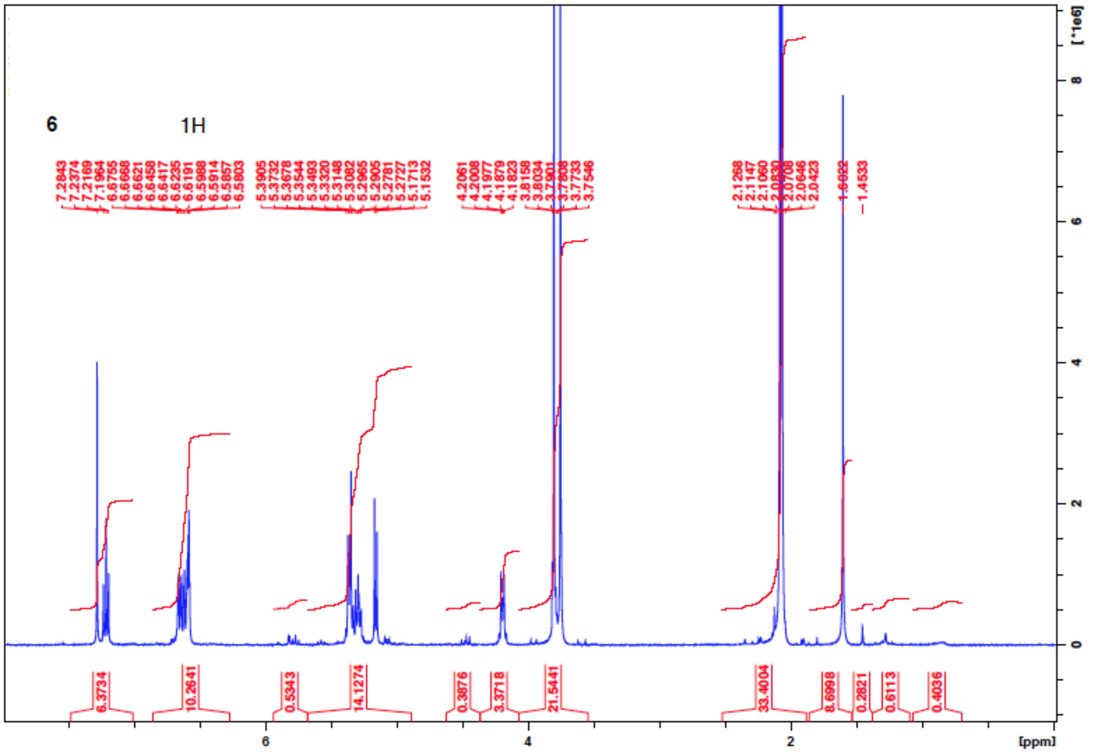

Figure S16.

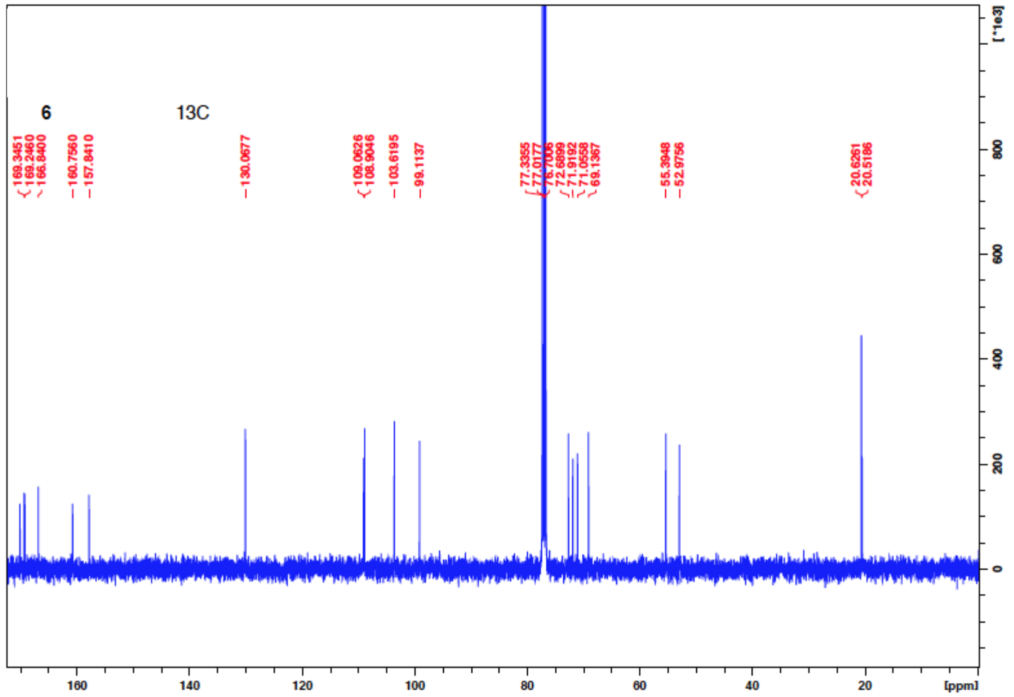

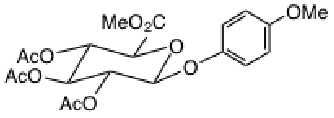

Figure S17. **Compound 7**

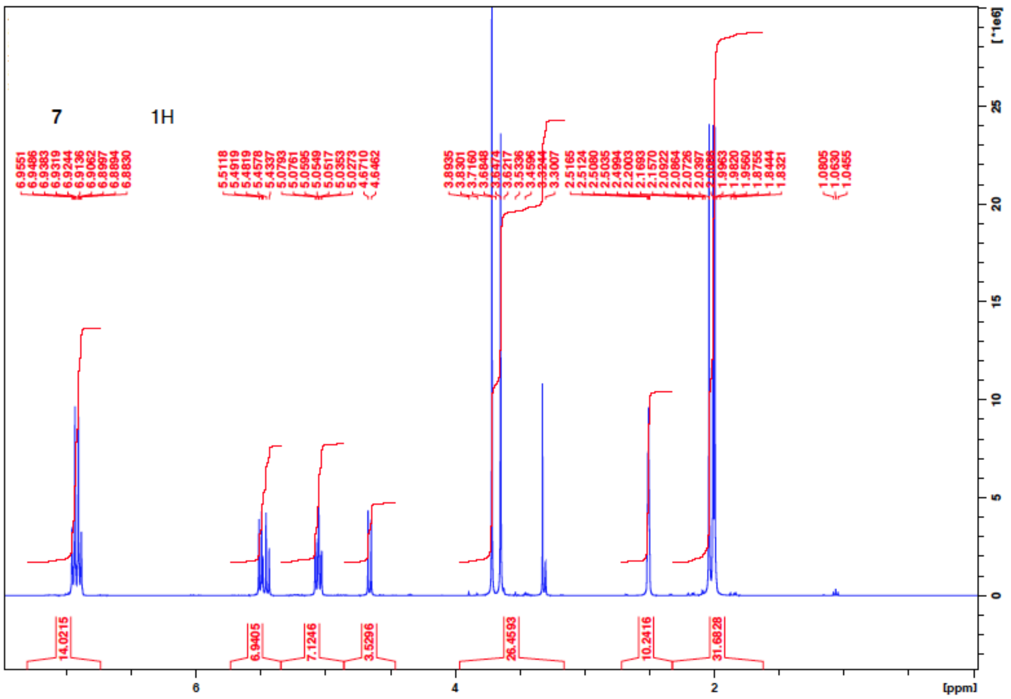

Figure S18.

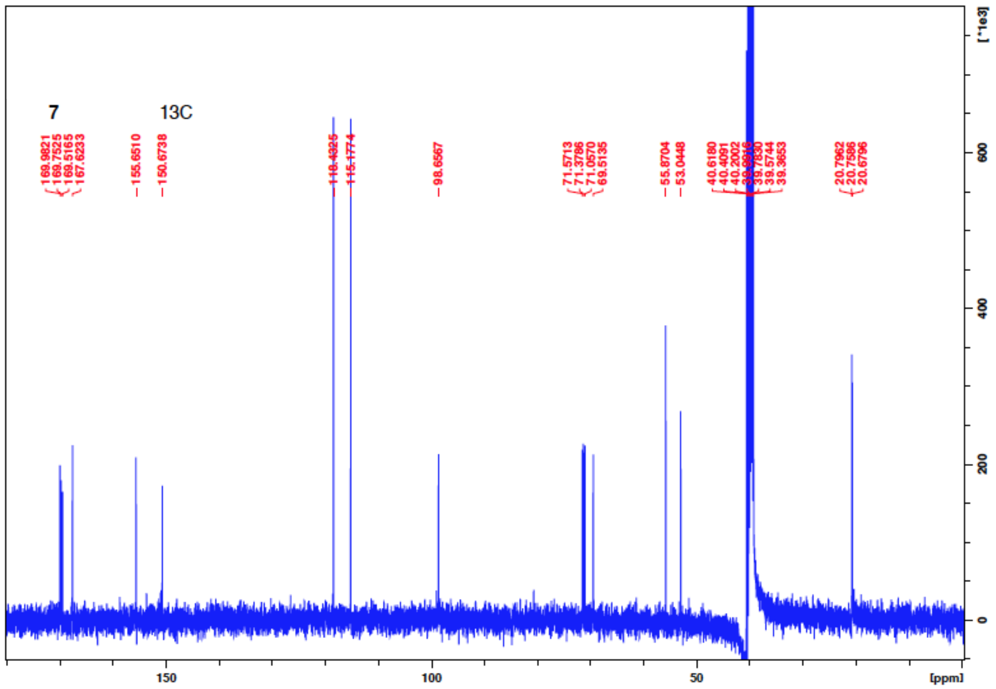

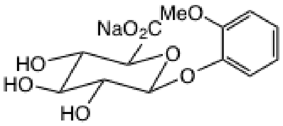

Figure S19. Compound 8

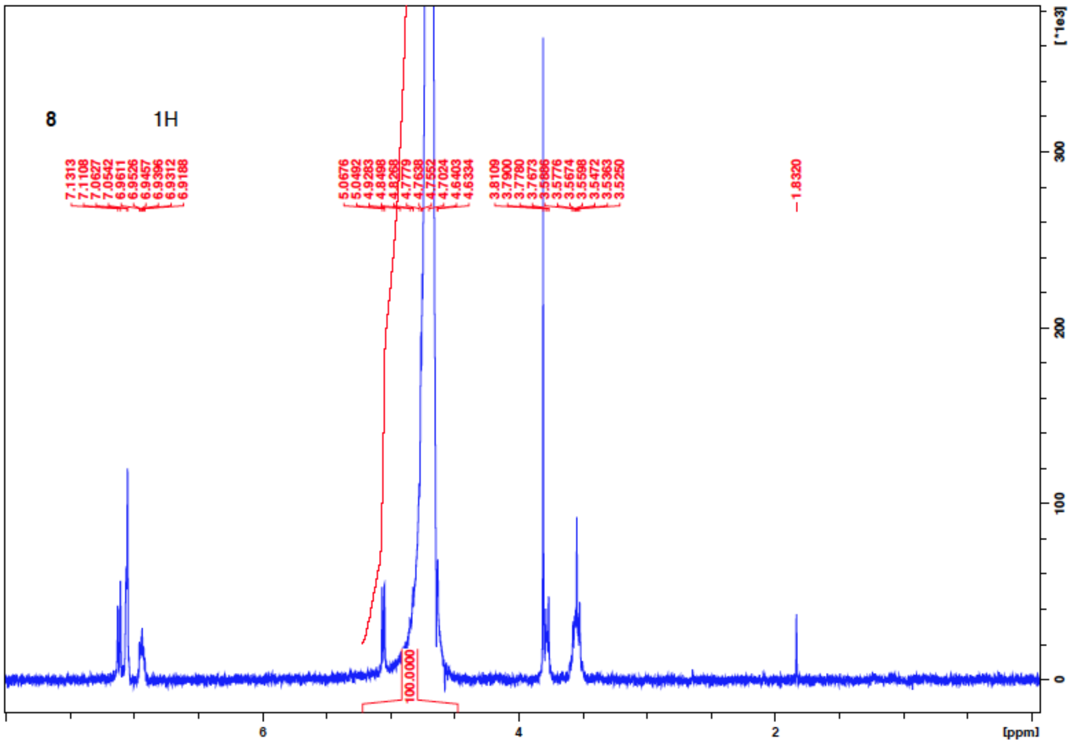

Figure S20.

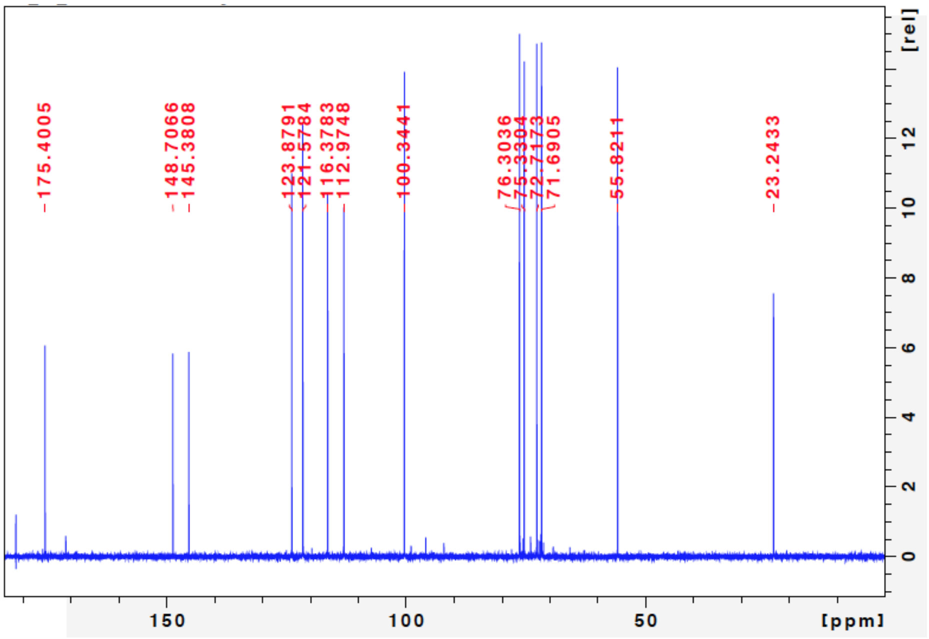

Signals at 1.8 ppm and 23.24 ppm (1H and 13C) are from acetate ions.

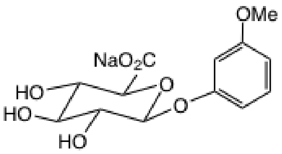

Figure S21. **Compound 9**

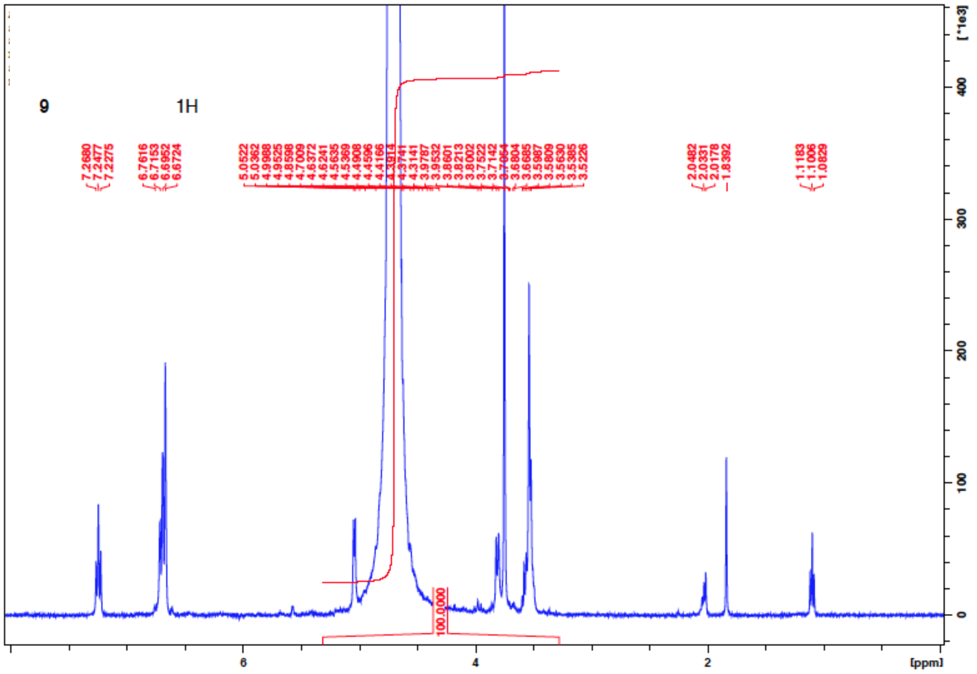

Figure S22.

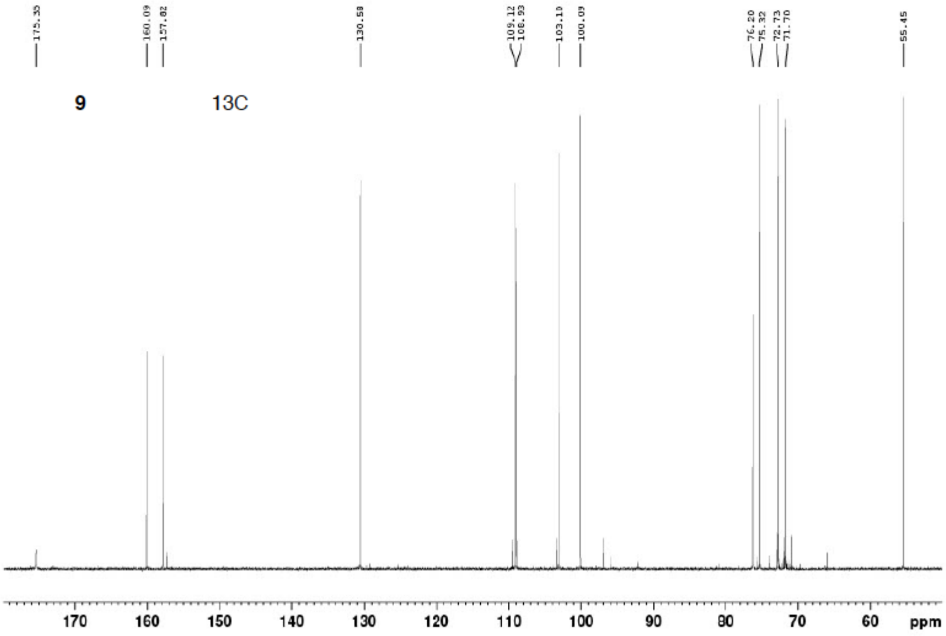

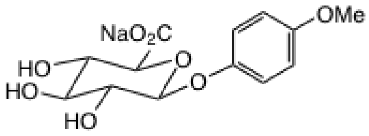

Figure S23. Compound 10

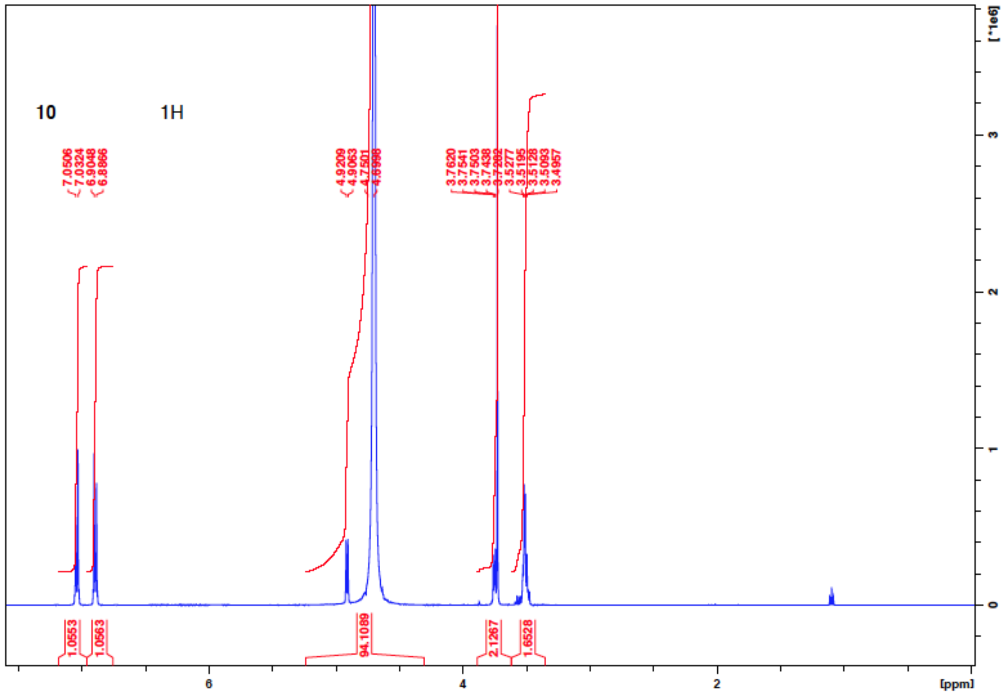

Figure S24.

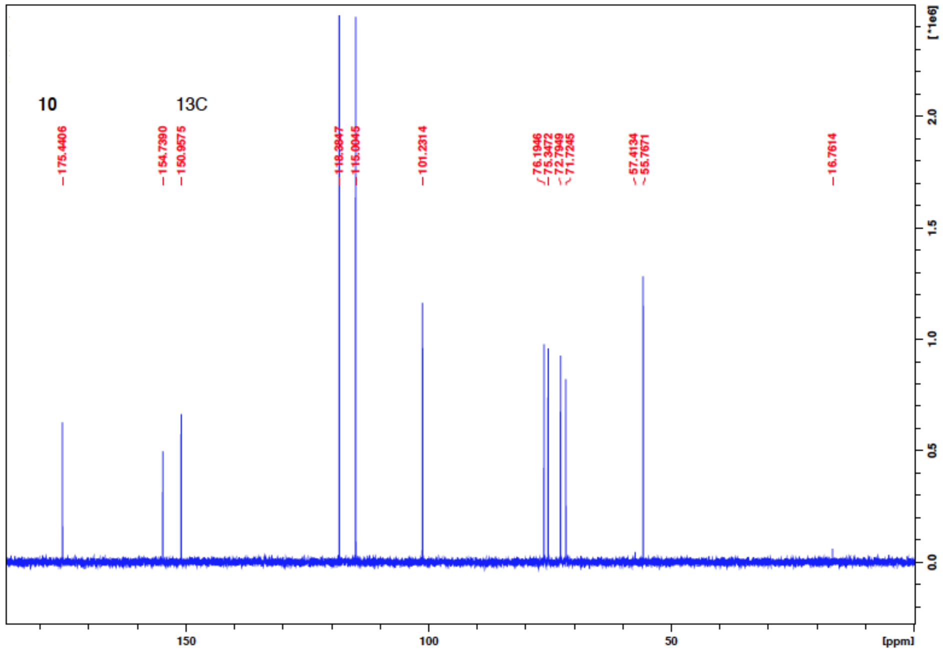

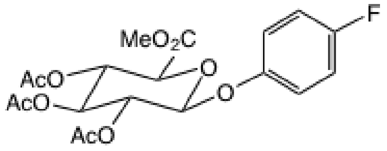

Figure S25. **Compound 18**

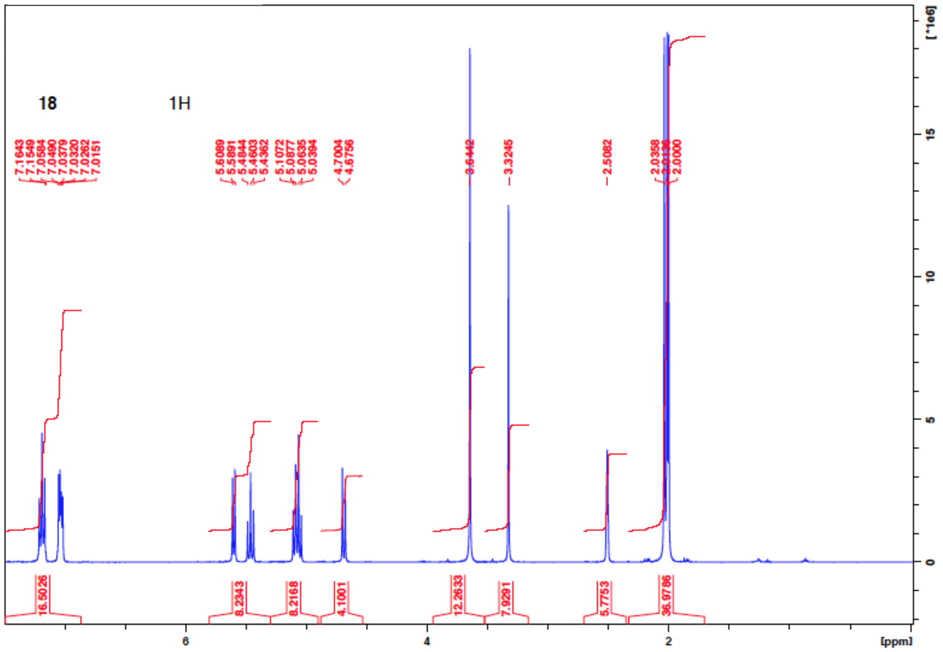

Figure S26.

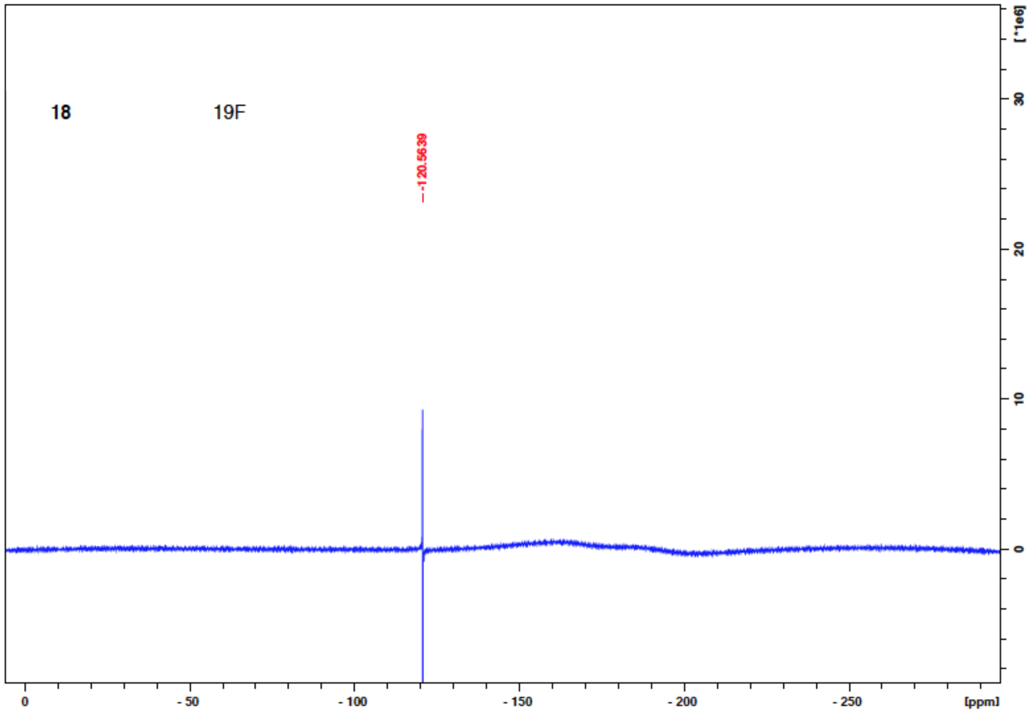

Figure S27

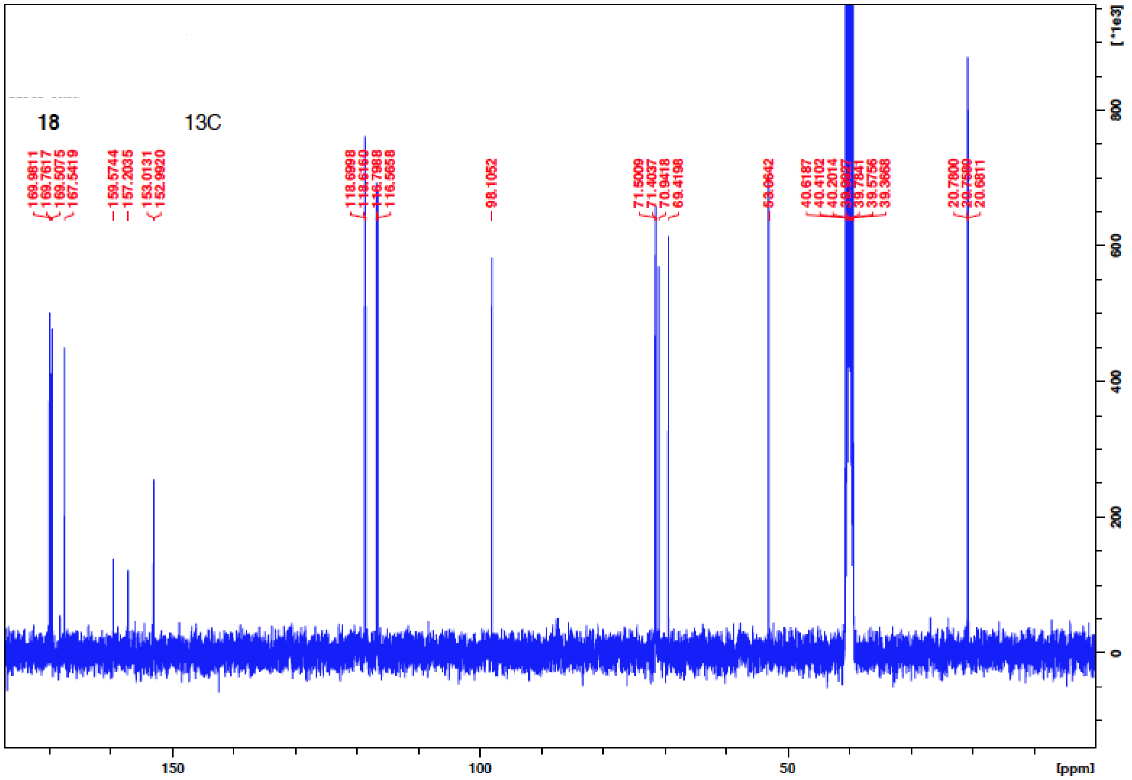

Figure S28. Compound 19.

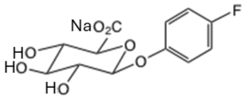

19

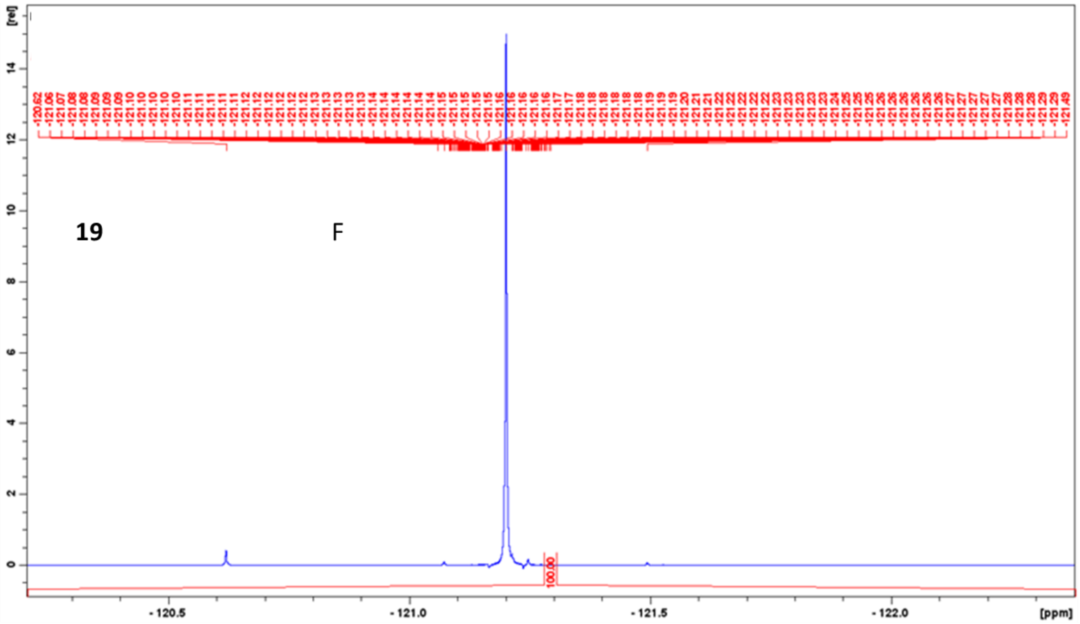

Figure S29.

19 H

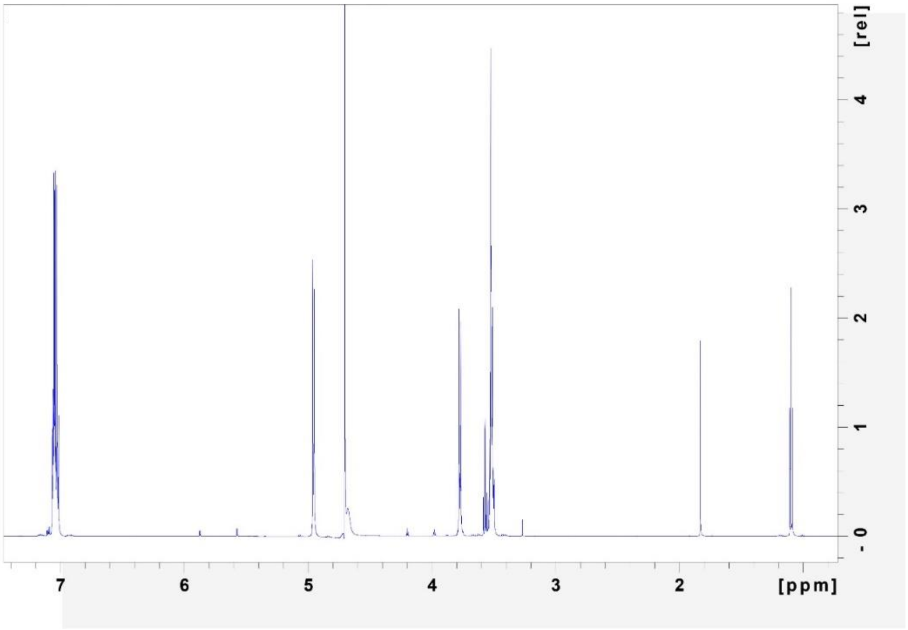

Figure S30

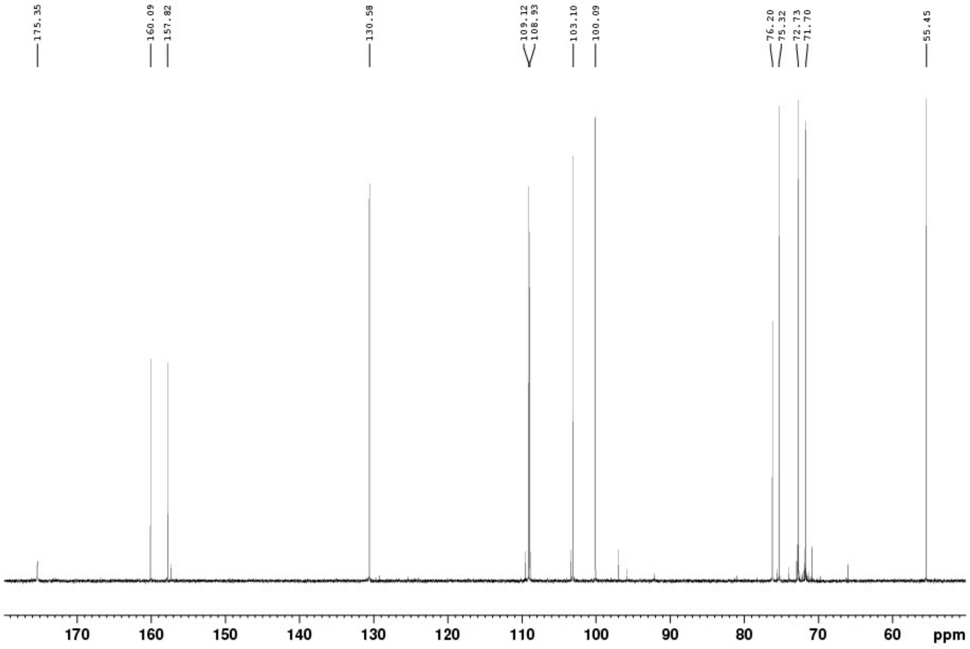

Figure S31.

***B. Caccae* with 2-methoxyphenol**

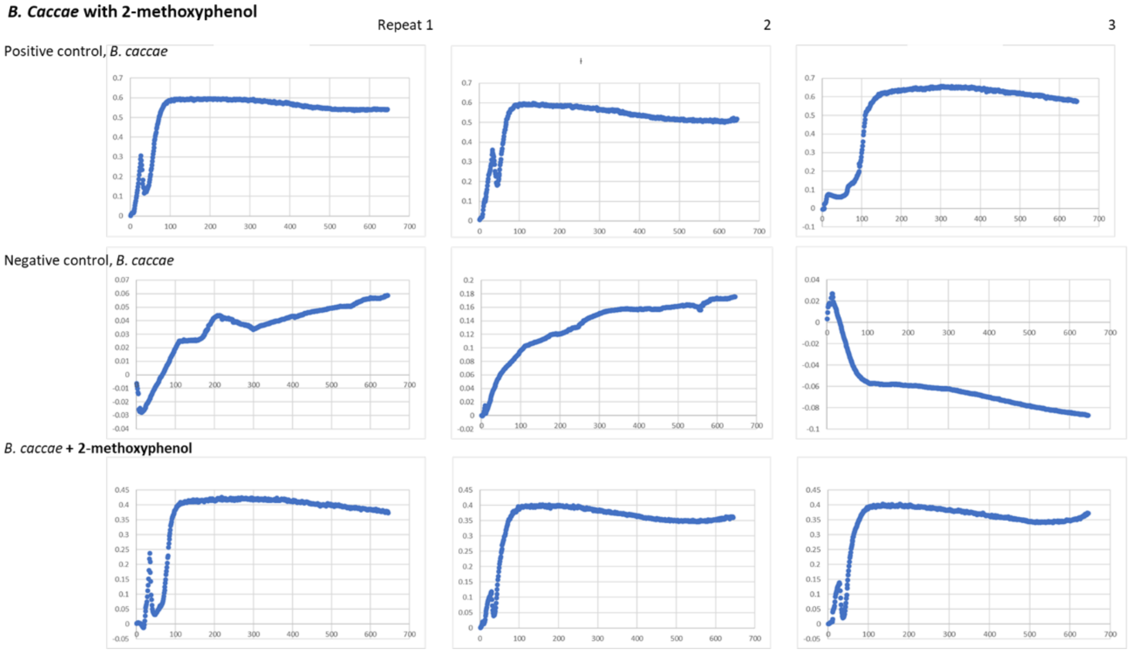

Figure S32.

***B. Caccae* with 2-methoxyphenyl glucuronide**

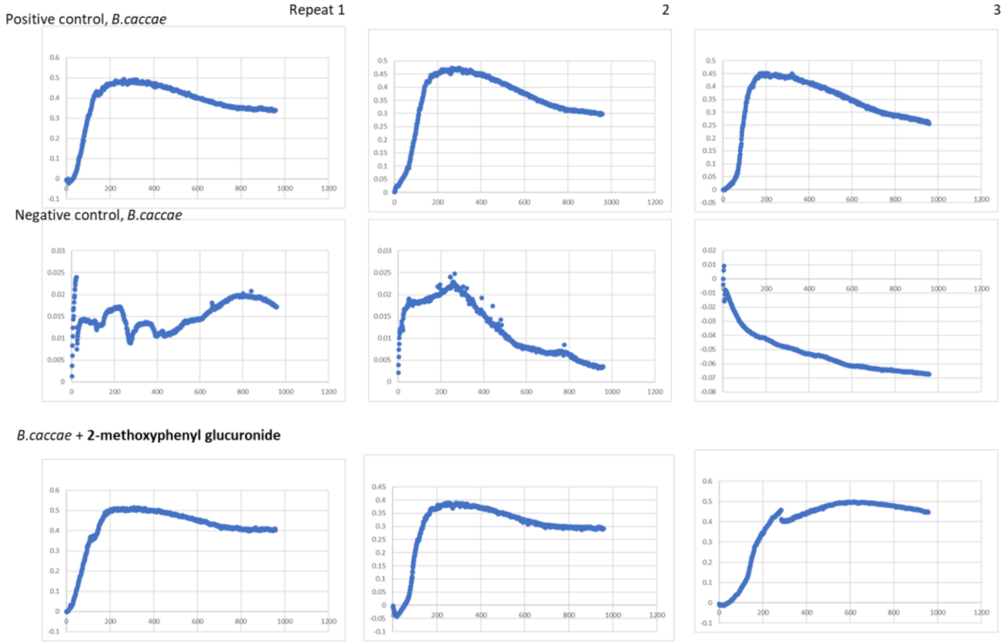

Figure S33.

***B. thetaiotaomicron* with 2-methoxyphenol**

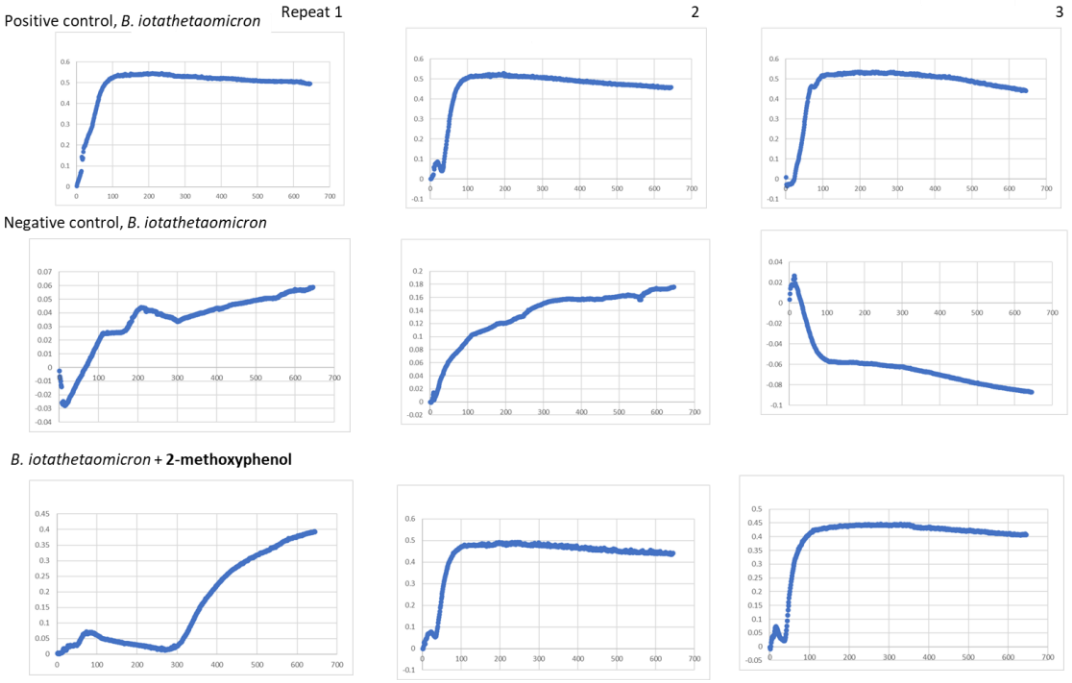

Figure S34.

***B. thetaiotaomicron* with 2-methoxyphenyl glucuronide**

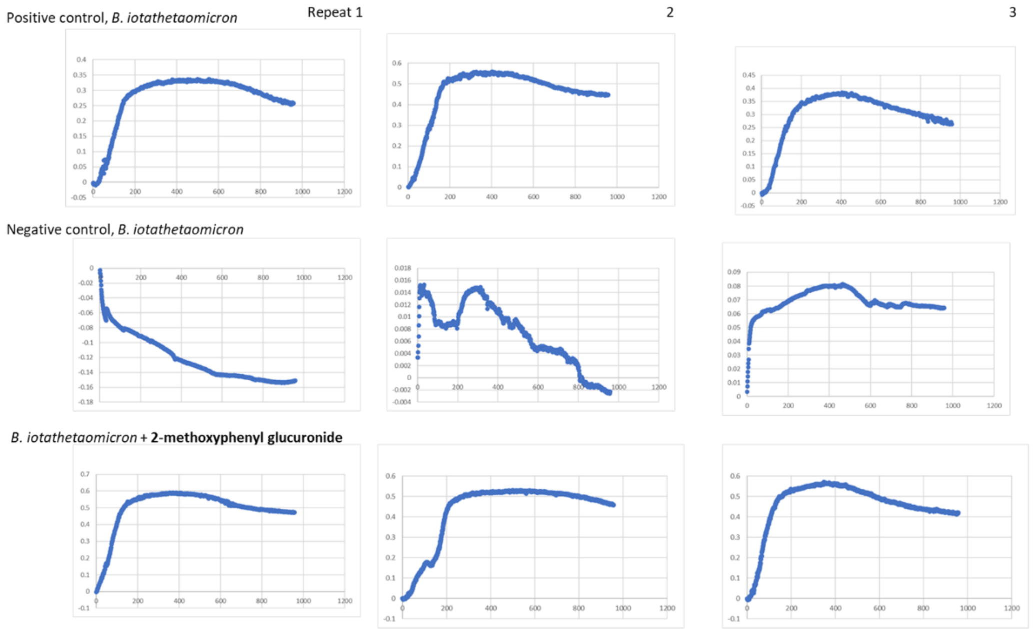

Figure S35.

***B. caccae* with resveratrol glucuronide**

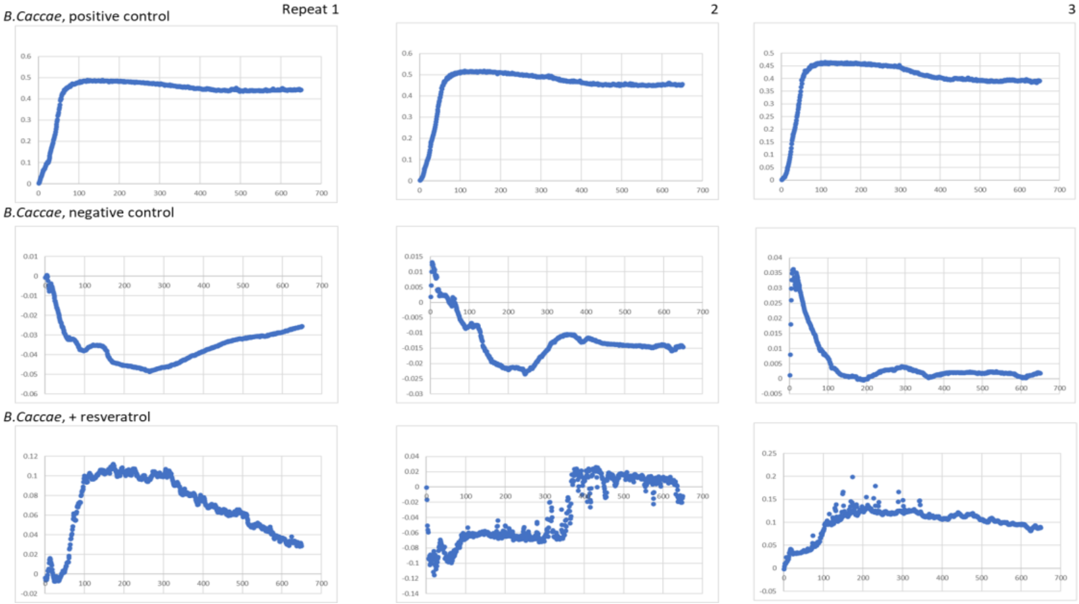

Figure S36.

***B. Caccae* with resveratrol glu**

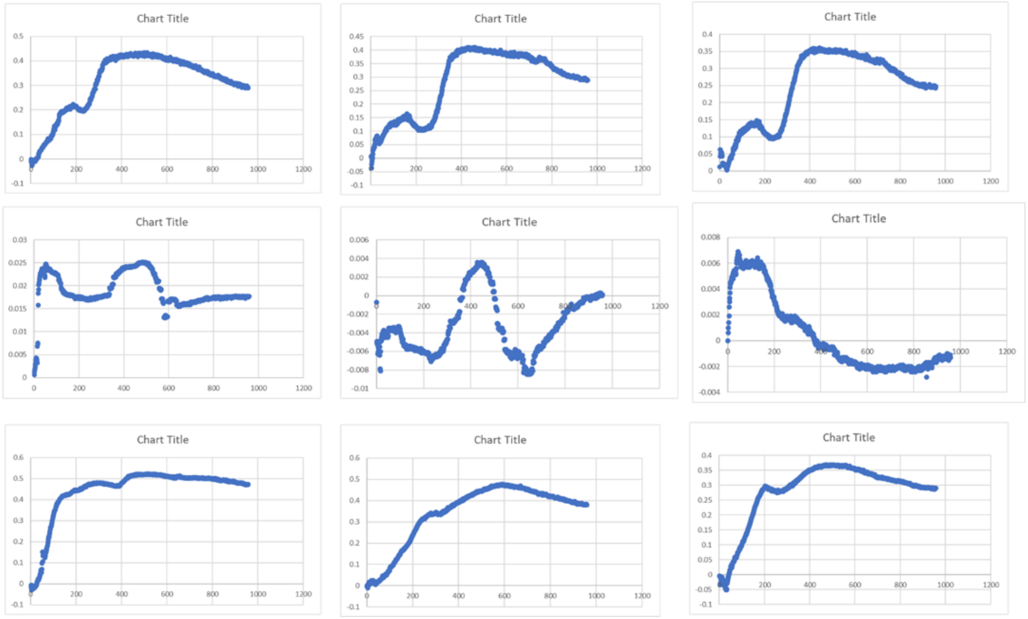

Figure S37.

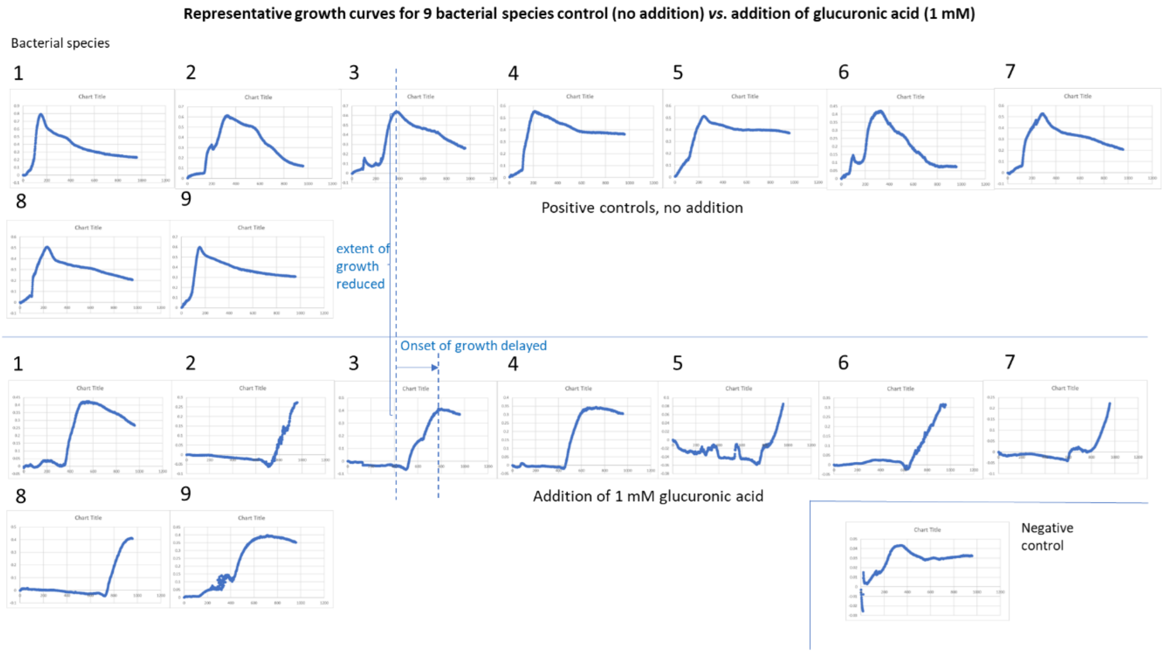
Representative growth curves for 9 bacterial species control (no addition) vs. addition of glucuronic acid (1 mM)
Bacterial species
1
2
3
4
5
6
7
8
9
Positive controls, no addition
extent of growth reduced
Onset of growth delayed
1
2
3
4
5
6
7
8
9
Addition of 1 mM glucuronic acid
Negative control
